# Synthesis of Novel (*S*)-3-(1-Aminoethyl)-8-pyrimidinyl-2-phenylisoquinolin-1(2*H*)-ones by Suzuki–Miyaura Coupling and Their Cell Toxicity Activities

**DOI:** 10.3390/ph15010064

**Published:** 2022-01-04

**Authors:** Ok Kyoung Choi, Yong Ho Sun, Hyemi Lee, Joon Kwang Lee, Tae Hoon Lee, Hakwon Kim

**Affiliations:** 1 Department of Applied Chemistry and Global Center for Pharmaceutical Ingredient Materials, Kyung Hee University, Yongin 17410, Korea; andante99@boryung.co.kr; 2 Process Research, Boryung R&D Institute, Ansan 15425, Korea; sunyh@boryung.co.kr (Y.H.S.); hmlee0325@samjinpharm.co.kr (H.L.); jklee@boryung.co.kr (J.K.L.)

**Keywords:** (S)-3-(1-aminoethyl)-8-pyrimidinyl-2-phenylisoquinoline-1(2H)-one, Suzuki–Miyaura coupling, antitumor, cytotoxicity

## Abstract

A series of (*S*)-3-(1-aminoethyl)-8-pyrimidinyl-2-phenylisoquinoline-1(2*H*)-ones **3a**–**3k** was synthesized in 40–98% yield through Suzuki–Miyaura coupling using Pd(PPh_3_)_2_Cl_2_, Sphos, and K_2_CO_3_ in THF/H_2_O mixed solvent. All newly synthesized compounds were evaluated for cell viability (IC_50_) against MDA-MB-231, HeLa, and HepG2 cells. The antitumor activities of **3a**–**3k** were improved when various pyrimidine motifs were introduced at position C-8 of the isoquinolinone ring.

## 1. Introduction

**Figure 1:**
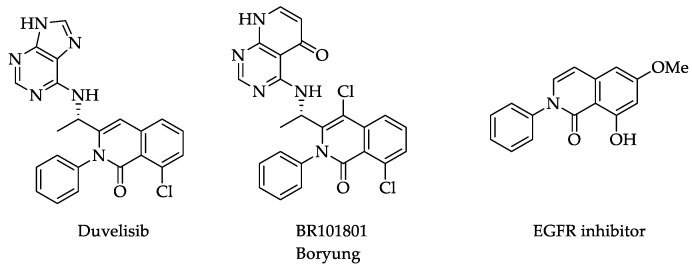
Examples of anti-cancer drugs containing the 2-phenylisoqunolin-1(2*H*)-one.

Isoquinolin-1(2*H*)-one derivatives are heterocyclic compounds exhibiting various bioactivities [1]. For example, the isoquinolin-1(2*H*)-one structure is known as an important pharmacophore of an effective 5-HT3 antagonist [2] and an inhibitor for the production of tumor necrosis factor α (TNF-α) [3]. In particular, 2-phenylisoquinolin-1(2*H*)-one has been reported as the main structure of several anticancer drugs that have been recently developed [4,5,6,7]. Duvelisib, which is used for the treatment of chronic lymphocytic leukemia (CLL) and somatic cell lymphoma (SLL), contains 2-phenylisoquinolin-1(2*H*)-one as a basic structure [6]. BR1018018, which has recently been developed as a PI3K selective inhibitor by Boryung Pharmaceutical (Korea), also has a 2-phenylisoquinoline-1(2*H*)-one moiety (Figure 1) [7].

**Figure 2:**
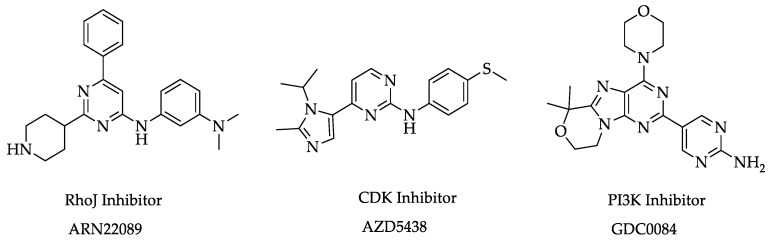
Candidates for anticancer drugs with pyrimidine cores.

On the other hand, 2-substituted pyrimidines consist of the basic structure of several drugs with various pharmacological activities. Recently, 2-substituted pyrimidines in RhoJ (Rho-related GTP-binding protein) inhibitor [8], CDK (Cyclin-dependent kinase) inhibitor [9], and PI3K (phosphoinositide 3-kinase) inhibitor [10] are important basic structures (Figure 2).

**Scheme 1:**
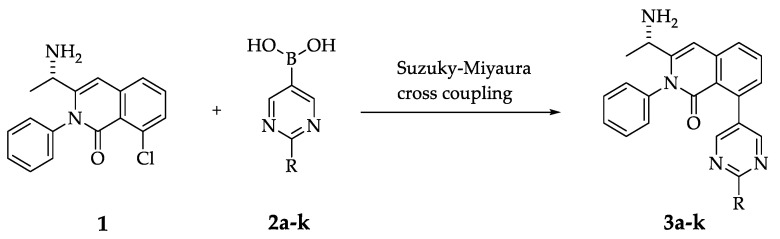
Synthesis of novel pyrimidinyl-2-phenylisoquinolin-1(2*H*)-ones **3** using SMC.

Based on these previous reports, we have become interested in whether 8-pyrimidinyl-2-phenylisoquinolin-1(*2H*)-one derivatives synthesized by the combination of 2-phenylisoquinolin-1(2*H*)-one and pyrimidine can be used as a new pharmacophore. Therefore, we investigated the synthesis of new (*S*)-3-(1-aminoethyl)-8-pyrimidinyl-2-phenylisoquinolin-1(2*H*)-one derivatives (**3**) by the Suzuki–Miyaura coupling (SMC) of (*S*)-3-(1-aminoethyl)-8-chloro-2-phenylisoquinolin-1(2*H*)-one (**1**) with various pyrimidinyl boronic acids **2** (Scheme 1). SMC is well known as the most common method for forming new carbon–carbon bonds in organic synthesis chemistry [11,12,13]. Well-known highly efficient SMC reactions, such as those using microwave chemistry, can be simply performed in a laboratory to produce a small amount of product by the column chromatography separation method [14]. However, there is a need to develop a mild, general, and practical SMC reaction capable of the bulk synthesis required in drug discovery research. Herein, we report an effective and mild synthesis method for (*S*)-3-(1-aminoethyl)-8-pyrimidinyl-2-phenylisoquinolin-1(2*H*)-one derivatives (**3**) and their anticancer activities.

## 2. Results and Discussion

### 2.1. Synthesis of Compounds

First, we compared the SMC reaction efficiencies of (*S*)-3-(1-aminoethyl)-8-chloro-2-phenylisoquinolin-1(2*H*)-one (**1**) prepared in a known method [15] with (2-methoxypyrimidin-5-yl)boronic acid (**2a**) on various Pd catalysts in dioxane/H_2_O for 12 h at 80 °C with reference to a previously reported mixed solvent system [16,17]. To screen Pd catalysts in SMC reactions, the synthesis efficiencies of **3a** for various Pd catalyst systems were compared under the above reaction conditions, including 5 mol% of Pd complex and 10 mol% of ligand (Table 1, Entries 1–5). When Pd(PPh_3_)_2_Cl_2_ and PPh_3_ (Entry 5) were used, **3a** was obtained in a low yield of 39%. Lower yields were also observed when using other common catalysts, such as Pd(PPh_3_)_4_, Pd(OAc)_2_/PPh_3_, or PdCl_2_/PPh_3_.

SMC reactions with Pd(PPh_3_)_2_Cl_2_ (2.5 mol%), selected as a Pd catalyst, and various phosphine ligands (5 mol%) were studied under conditions in which the amount of catalyst used was reduced by half of that given above. First, the reaction efficiency was investigated by increasing the bulkiness of the ligand using mono-phosphine ligands (PPh_3_ < P(O-tol)_3_ < P(Cy)_3_) (Entries 6–8) and di-phosphine ligands (dppf < Xantphos) (Entries 9,11). It was observed that mono-phosphine ligands or di-phosphine ligands [18] showed low reactivity (Entries 1–9,11). However, the yield increased in the electron-rich phosphine ligands, such as Aphos (Entry 10). Unlike the mono-aryl type Aphos, the yield as well as the reaction rate increased dramatically when using Buchwald ligands, such as Sphos, Ruphos, and Davephos, which are biaryl types (Entries 13–15). We attributed this to the structural characteristics of the electron-rich and bulky phosphine ligands substituted with cyclohexyl or biaryl groups. This is consistent with reports that electron-rich and bulky phosphine Buchwald ligands accelerate reductive elimination and oxidative addition in SMC reactions, resulting in increased reactivity [19,20,21].

Subsequently, base screening with various solvents was performed in SMC (Table 2).

The SMC reaction occurred in the highest yield in the THF/H_2_O mixed solvent system. In addition, phase separation occurred in the THF/H_2_O solvent system after the reaction was completed, but phase separation was not observed in the aqueous solution system mixed with other solvents. Therefore, in this solvent system, the separated organic layer was extracted with an acidic aqueous solution, neutralized, and then crystallized to easily obtain a high-purity product without a chromatographic separation, which is very advantageous for a large-scale synthesis process. To further develop this process, a design of experiments (DoE; using Design Expert 12) was performed and optimized. The optimal conditions established through the DoE were Pd(PPh_3_)_2_Cl_2_ (0.5 mol%), Sphos (1.5 mol%), and K_2_CO_3_ (3.0 eq) in THF and H_2_O mixed solvent. It was found that the amount of solvent THF/H_2_O used to facilitate phase separation between the organic layer and the aqueous layer after the reaction was more than 10 mL per gram of substrate **1**.

Novel (*S*)-3-(1-aminoethyl)-8-pyrimidinyl-2-phenylisoquinolin-1(2*H*)-one derivatives (**3**) were synthesized by using SMC reactions under these optimized conditions (Table 3). The structure of **3a**–**3k** were confirmed by ^1^H-NMR, ^13^C-NMR spectrosco-py (Supplementary Materials Figures S1–S22). In the case of unstable boronic acid, pinacol boronate (in case of **3g** and **3k**) was used as a reactant. Electron-rich pyrimidinyl boronic acid substituted with methoxy, ethoxy, dimethylamino, and piperidinyl groups at the para position of the pyrimidine ring gave the product in good yield (**3a**, **3b**, **3i** and **3j**). In the case of other boronic acids with low reactivity, the reaction could be completed in good yield by increasing the amount of catalyst and ligand (**3c**, **3e**–**3g**). Boronic acids with steric hindrance, such as 2,4-dimethoxypyrimidinyl boronic acid, still showed low yield even when the amount of catalyst and ligand was increased (**3d**). In addition, electron-deficient boronic acid (e.g., 2-cyanopyrimidinyl boronic acid) showed the lowest reactivity (**3k**).

### 2.2. Anti-Cancer Activity

All newly synthesized compounds (**3a**–**3k**) were evaluated for cell viability (IC_50_) against MDA-MB-231 (metallic breast cancer cells), HeLa (cervical epithelial carcinoma cell), and HepG2 (hepatic carcinoma cell) to evaluate the effect of different pyrimidinyl groups on (*S*)-3-(1-aminoethyl)-8-chloro-2-phenylisoquinolin-1(2*H*)-one **1** (Table 4). The cell toxicity data of compounds **1**, **3a**–**3k** for MDA-MB 231, HeLa, HepG2 were de-scribed in Supplementary Materials Figures S23–S34.

Compound **1** without a pyrimidine substituent showed less than 50% cell death in MDA-MB-231, HeLa, and HepG2 cells at a concentration of 10 μM, so its IC_50_ value could not be determined. However, compound **3** substituted with various pyrimidines showed excellent cytotoxic activity in tumor cell lines. Since in MDA-MB-231 simple pyrimidinyl (**3e**), 4-methylpyrimidinyl (**3f**), 4-methoxypyrimidinyl (**3a**), or 2,4-dimethoxypyrimidinyl (**3d**) compounds had no cytotoxic activity, their IC_50_ values could not be determined. On the other hand, 4-alkoxypyrimidinyl (**3b**,**3c**), 4-aminopyrimidinyl (**3h**,**3i**), 4-piperidinopyrimidinyl (**3j**), and 4-cyanopyrimidinyl (**3k**) compounds showed strong anticancer activity. In particular, the cytotoxic activity increased as the alkyl chain of the alkoxy substituent in the 4-alkoxypyrimidinyl compounds increased (**3a** < **3b**,**3c**). These results indicate that the cytotoxic activity of the novel synthetic 8-pyrimidinylisoquinolinones **3a**–**3k** on cancer cells was slightly different depending on the type of cell. Although cell viability assays, such as the MTT assay, alone cannot fully explain these results, overall cytotoxicity appears to be dependent on the polarity of the compound. In HeLa and HepG2 cells, all pyrimidine-substituted isoquinolinones **3a**–**3k** showed excellent cytotoxic activity. Although all compounds showed good cytotoxic activity, 4-alkoxypyrimidinyl (**3b**, **3c**), 4-aminopyrimidinyl (**3h**, **3i**), 4-piperidinopyrimidinyl (**3j**), and 4-cyanopyrimidinyl (**3k**) compounds showed higher activity than others. In particular, compound **3k**, which has an electron-withdrawing group (CN) in a pyrimidine ring, showed the lowest IC_50_ value under this condition.

## 3. Materials and Methods

### 3.1. General

All commercially available materials from Sigma-Aldrich (Burlington, MA, USA), Daejung (Siheung, Korea), TCI (Tokyo, Japan), Chemieliva (Chongqing, China) and solvents were used without further purification. All small-scale screening reactions (≤10 mL of solvent) were performed in 50 mL round bottom flasks on a Radleys Carousel 6 Plus Reaction Station under an air atmosphere. HPLC was performed on a Hitachi LaChrom Elite HPLC system. ^1^H NMR (400 MHz) and ^13^C NMR (100 MHz) were measured on a Bruker Avance 400 spectrometer system. ^1^H NMR spectra chemical shifts were expressed in parts per million (ppm) downfield from tetramethylsilane, and coupling constants were reported in Hertz (Hz). Splitting patterns are indicated as follows: s, singlet; d, doublet; t, triplet; and q, quartet; m, multiplet. ^13^C NMR spectra were reported in ppm, referenced to deuterochloroform (77.16 ppm). Melting points were determined by DSC (Mettler Toledo). High resolution mass spectra (HRMS, JEOL MStation JMS-700) were obtained using an electron impact (EI) ionization technique (magnetic sector—electric sector double focusing mass analyzer) from the KBSI (Korea Basic Science Institute Daegu Center).

### 3.2. General Procedure for the Suzuki-Miyaura Coupling Reactions (***3a**~**3k***)

Suzuki–Miyaura coupling reactions (Table 3) were typically performed on a 1.67 mmol scale of the aryl halide **1**. A mixture of (*S*)-3-(1-aminoethyl)-8-chloro-2-phenylisoquinolin-1(2*H*)-one **1** (0.5 g, 1.67 mmol), 2-substituted pyrimidinyl boronic acid or 2-substituted pyrimidinyl pinacol boronate **2** (1.2 equiv, 2 mmol), K_2_CO_3_ (0.3 g, 2.17 mmol), Pd(PPh_3_)_2_Cl_2_ (0.50–5 mol%), and Sphos (1.5–15 mol%) in mixed solvent (THF 5 mL, water 5 mL) was stirred at 65 °C in 50 mL round bottom flasks for 12 h. After the biphasic reaction solution was cooled to room temperature, the organic layer was separated. The organic layer was concentrated under vacuum and diluted with CH_2_Cl_2_ (20 mL) and 0.3 N HCl (20 mL). The aqueous layer was separated and washed with CH_2_Cl_2_ (20 mL). The aqueous layer was basified with aqueous NH_4_OH (1 mL) and extracted two times with CH_2_Cl_2_ (2 × 20 mL). The organic phase was concentrated under vacuum. Further purification of product was isolated from a flash chromatography using silica gel (300–400 mesh) with CH_2_Cl_2_–methanol as an eluent.

3-[(1*S*)-1-aminoethyl]-8-(2-methoxypyrimidin-5-yl)-2-phenyl-isoquinolin-1-one (**3a**). White solid, ${\left[\alpha \right]}_{D}^{20}$ −1.33 (c 0.01, CH_3_CN), mp 210–214 °C. 610 mg, 98% yield. ^1^H NMR (400 MHz, CDCl_3_) δ 8.45 (s, 2H), 7.62–7.70 (m, 2H), 7.47–7.51 (m, 2H), 7.40–7.44 (m, 1H), 7.19–7.23 (m, 3H), 6.84 (s, 1H), 3.98 (s, 1H), 3.69 (q, J = 6.5 Hz, 1H), 1.53 (s, 2H), 1.28 (d, J = 6.5 Hz, 3H). ^13^C NMR (100 MHz, CDCl_3_) δ 164.3, 162.9, 157.9, 149.8, 139.0, 138.1, 137.2, 132.0, 130.5, 130.0, 129.8, 129.7, 129.2, 128.9, 128.7, 127.3, 122.1, 102.1, 54.8, 54.8, 46.9, 23.7 HRMS (EI^+^): *m*/*z* [M + H] + calcd for C_22_H_20_N_4_O_2_ 372.1586, found 372.1584.

3-[(1*S*)-1-aminoethyl]-8-(2-ethoxypyrimidin-5-yl)-2-phenyl-isoquinolin-1-one (**3b**). White solid, ${\left[\alpha \right]}_{D}^{20}$ −3.51 (c 0.01, CH_3_CN), mp 185–188 °C. 629 mg, 97% yield. ^1^H NMR (400 MHz, CDCl_3_) δ 8.44 (s, 2H), 7.60–7.69 (m, 2H), 7.47–7.51 (m, 2H), 7.40–7.44 (m, 1H), 7.19–7.23 (m, 3H), 6.83 (s, 1H), 4.39 (q, J = 7.1 Hz, 2H), 3.69 (q, J = 6.5 Hz, 1H), 1.41 (t, J = 7.1 Hz, 3H), 1.34 (s, 2H), 1.28 (d, J = 6.5 Hz, 3H). ^13^C NMR (100 MHz, CDCl_3_) δ 164.0, 162.9, 157.90, 149.8, 139.0, 138.1, 137.4, 132.0, 130.5, 129.8, 129.7, 129.3, 128.9, 128.7, 127.2, 122.1, 102.1, 63.3, 46.9, 23.7, 14.6 HRMS (EI^+^): *m*/*z* [M + H] + calcd for C_23_H_22_N_4_O_2_ 386.1743, found 386.1743.

3-[(1*S*)-1-aminoethyl]-8-(2-isopropoxypyrimidin-5-yl)-2-phenyl-isoquinolin-1-one (**3c**). White solid, ${\left[\alpha \right]}_{D}^{20}$ −0.99 (c 0.01, CH_3_CN), mp 142–148 °C. 638 mg, 95% yield. ^1^H NMR (400 MHz, CDCl_3_) δ 8.43 (s, 2H), 7.62–7.70 (m, 2H), 7.47–7.51 (m, 2H), 7.40–7.44 (m, 1H), 7.19–7.23 (m, 3H), 6.83 (s, 1H), 5.25(m, J = 6.2 Hz, 1H), 3.70 (q, J = 6.5 Hz, 1H), 1.52 (s, 2H), 1.37 (d, J = 6.2 Hz, 6H), 1.28 (d, J = 6.5 Hz, 3H). ^13^C NMR (100 MHz, CDCl_3_) δ 163.6, 163.0, 158.0, 149.7, 139.0, 138.2, 137.5, 132.0, 130.6, 129.9, 129.8, 129.6, 129.3, 128.9, 128.8, 127.2, 122.1, 102.1, 70.0, 46.9, 23.7, 22.1 HRMS (EI^+^): *m*/*z* [M + H] + calcd for C_24_H_24_N_4_O_2_ 400.1899, found 400.1902.

3-[(1*S*)-1-aminoethyl]-8-(2,4-dimethoxypyrimidin-5-yl)-2-phenyl-isoquinolin-1-one (**3d**). White solid, ${\left[\alpha \right]}_{D}^{20}$ −1.82 (c 0.01, CH_3_CN), 303 mg, 45% yield. ^1^H NMR (400 MHz, CDCl_3_) δ 8.04 (s, 1H), 7.65 (m, 1H), 7.58 (m, 1H), 7.43–7.50 (m, 3H), 7.19 (m, 3H), 6.79 (s, 0.5H), 6.75 (s, 0.5H), 3.95 (s, 3H), 3.87 (s, 0.5H), 3.85 (s, 0.5H), 3.67 (m, J = 6.3 Hz, 1H), 1.44 (s, 2H), 1.30 (d, J = 6.4 Hz, 1.5H), 1.26 (d, J = 6.4 Hz, 1.5H). ^13^C NMR (100 MHz, CDCl_3_) δ 168.6, 164.6, 162.8, 184.7, 154.6, 149.4, 149.3, 138.5, 138.4, 135.4, 132.1, 130.0, 130.0, 129.9, 129.8, 129.4, 129.2, 129.0, 128.9, 128.7, 127.0, 123.6, 123.5, 118.8, 102.0, 101.9, 54.7, 53.9, 47.0, 46.9, 23.8, 23.4 HRMS (EI^+^): *m*/*z* [M + H] + calcd for C_23_H_22_N_4_O_3_ 402.1692, found 402.1689.

3-[(1*S*)-1-aminoethyl]-2-phenyl-8-pyrimidin-5-yl-isoquinolin-1-one (**3e**). White solid, ${\left[\alpha \right]}_{D}^{20}$ −2.24 (c 0.01, CH_3_CN), mp 205–209 °C. 522 mg, 91% yield. ^1^H NMR (400 MHz, CDCl3) δ 9.08 (s, 1H), 8.68 (s, 2H), 7.66–7.73 (m, 2H), 7.47–7.51 (m, 2H), 7.40–7.44 (m, 1H), 7.19–7.22 (m, 3H), 6.86 (s, 1H), 3.70 (q, J = 6.5 Hz, 1H), 2.12 (s, 2H), 1.28 (d, J = 6.5 Hz, 3H). ^13^C NMR (100 MHz, CDCl_3_) δ 162.8, 156.7, 155.6, 150.1, 139.0, 138.0, 137.0, 136.7, 132.0, 130.4, 129.9, 129.8, 129.2, 128.9, 128.7, 127.7, 122.1, 102.1, 46.9, 23.7 HRMS (EI^+^): *m*/*z* [M + H] + calcd for C_21_H_18_N_4_O 342.1481, found 342.1484.

3-[(1*S*)-1-aminoethyl]-8-(2-methylpyrimidin-5-yl)-2-phenyl-isoquinolin-1-one (**3f**). White solid, ${\left[\alpha \right]}_{D}^{20}$ −1.55 (c 0.01, CH_3_CN), mp 221–237 °C. 567 mg, 95% yield. ^1^H NMR (400 MHz, CDCl_3_) δ 8.58 (s, 2H), 7.63–7.71 (m, 2H), 7.46–7.50 (m, 2H), 7.39–7.43 (m, 1H), 7.19–7.22 (m, 3H), 6.84 (s, 1H), 3.70 (q, J = 6.5 Hz, 1H), 2.71 (s, 3H), 1.50 (s, 2H), 1.28 (d, J = 6.5 Hz, 3H). ^13^C NMR (100 MHz, CDCl_3_) δ 165.7, 162.84, 155.6, 149.9, 139.0, 138.1, 137.3, 133.3, 13, 130.4, 129.8, 129.7, 129.2, 128.9, 128.7, 127.5, 122.1, 102.0, 46.9, 25.8, 23.7 HRMS (EI^+^): *m*/*z* [M + H] + calcd for C_22_H_20_N_4_O 356.1637, found 356.1639.

3-[(1*S*)-1-aminoethyl]-8-[2-(1-hydroxy-1-methyl-ethyl)pyrimidin-5-yl]-2-phenyl-isoquinolin-1-one (**3g**). White solid, ${\left[\alpha \right]}_{D}^{20}$ −2.44 (c 0.01, CH_3_CN), mp 216–220 °C. 629 mg, 94% yield. ^1^H NMR (400 MHz, CDCl_3_) δ 8.68 (s, 2H), 7.68–7.75 (m, 2H), 7.50–7.52 (m, 2H), 7.44–7.47 (m, 1H), 7.21–7.24 (m, 3H), 6.88 (s, 1H), 3.71 (q, J = 6.5 Hz, 1H), 1.62 (s, 6H), 1.30 (d, J = 6.5 Hz, 3H). ^13^C NMR (100 MHz, CDCl_3_) δ 171.8, 162.9, 155.6, 150.1, 139.1, 138.1, 137.0, 134.1, 132.1, 130.8, 129.9, 129.2, 129.1, 128.7, 127.8, 122.1, 102.2, 73.1, 46.9, 30.0, 23.8 HRMS (EI^+^): *m*/*z* [M + H] + calcd for C_24_H_24_N_4_O_2_ 400.1899, found 400.1898.

3-[(1*S*)-1-aminoethyl]-8-(2-aminopyrimidin-5-yl)-2-phenyl-isoquinolin-1-one (**3h**). White solid, ${\left[\alpha \right]}_{D}^{20}$ −1.83 (c 0.01, CH_3_CN), mp 242–247 °C. 365 mg, 61% yield. 1H NMR (400 MHz, CDCl_3_) δ 8.27 (s, 2H), 7.63–7.67 (m, 1H), 7.59–7.57 (m, 1H), 7.46–7.51 (m, 2H),7.40–7.43 (m, 1H), 7.19–7.23 (m, 3H), 6.80 (s, 1H), 5.11(s, 2H), 3.68 (q, J = 6.5 Hz, 1H), 1.47 (s, 2H), 1.27 (d, J = 6.5 Hz, 3H). ^13^C NMR (100 MHz, CDCl_3_) δ 163.0, 161.6, 157.2, 149.7, 139.0, 138.3, 138.2, 132.0, 130.4, 129.8, 129.7, 129.3, 128.8, 126.8, 126.6, 122.1, 102.1, 46.9, 23.7 HRMS (EI^+^): *m*/*z* [M + H] + calcd for C_21_H_19_N_5_O 357.1590, found 357.1593.

3-[(1*S*)-1-aminoethyl]-8-[2-(dimethylamino)pyrimidin-5-yl]-2-phenyl-isoquinolin-1-one (**3i**). White solid, ${\left[\alpha \right]}_{D}^{20}$ −7.03 (c 0.01, CH_3_CN), mp 244–248 °C. 563 mg, 87% yield. ^1^H NMR (400 MHz, CDCl_3_) δ 8.30 (s, 2H), 7.62–7.66 (m, 1H), 7.54–7.56 (m, 1H), 7.45–7.49 (m, 2H), 7.38–7.42 (m, 1H), 7.19–7.22 (m, 3H), 6.78 (s, 1H), 3.68 (q, J = 6.5 Hz, 1H), 3.16 (s, 6H), 1.52 (s, 2H), 1.27 (d, J = 6.5 Hz, 3H). ^13^C NMR (100 MHz, CDCl_3_) δ 163.0, 161.0, 156.7, 154.9, 149.4, 139.0, 138.9, 138.4, 132.0, 130.3, 129.7, 129.7, 129.4, 128.9, 128.7, 126.4, 123.6, 122.0, 102.0, 46.9, 37.3, 23.6 HRMS (EI^+^): *m*/*z* [M + H] + calcd for C_23_H_23_N_5_O 385.1903, found 385.1905.

3-[(1*S*)-1-aminoethyl]-2-phenyl-8-[2-(1-piperidyl)pyrimidin-5-yl]isoquinolin-1-one (**3j**). White solid, ${\left[\alpha \right]}_{D}^{20}$ −5.03 (c 0.01, CH_3_CN), 655 mg, 92% yield. ^1^H NMR (400 MHz, CDCl_3_) δ 8.28 (s, 2H), 7.62–7.65 (m, 1H), 7.53–7.56 (m, 1H), 7.46–7.50 (m, 2H), 7.39–7.43 (m, 1H), 7.19–7.23 (m, 3H), 6.78 (s, 1H), 3.76 (t, J = 4.9 Hz, 4H), 3.69 (q, J = 6.5 Hz, 1H), 1.58-1.64 (m, 8H), 1.27 (d, J = 6.5 Hz, 3H). ^13^C NMR (100 MHz, CDCl_3_) δ 163.0, 160.4, 156.8, 149.4, 139.0, 138.9, 138.4, 132.0, 130.0, 129.8, 129.7, 129.4, 128.9, 128.8, 126.4, 123.8, 122.0, 102.1, 46.9, 44.8, 25.9, 24.9, 23.6 HRMS (EI^+^): *m*/*z* [M + H] + calcd for C_26_H_27_N_5_O 425.2216, found 425.2212

5-[3-[(1*S*)-1-aminoethyl]-1-oxo-2-phenyl-8-isoquinolyl]pyrimidine-2-carbonitrile (**3k**). White solid, ${\left[\alpha \right]}_{D}^{20}$ −3.54 (c 0.01, CH_3_CN), 246 mg, 40% yield. ^1^H NMR (400 MHz, CDCl_3_) δ 8.74 (s, 2H), 7.74–7.75 (m, 2H), 7.42–7.52 (m, 3H), 7.20–7.21 (m, 3H), 6.91 (s, 1H), 3.71 (q, J = 6.3 Hz, 1H), 1.47 (s, 2H), 1.28 (d, J = 6.3 Hz, 3H). ^13^C NMR (100 MHz, CDCl_3_) δ 162.8, 156.5, 150.5, 142.7, 139.2, 139.2, 137.7, 135.2, 132.2, 130.1, 130.0, 129.9, 129.2, 129.0, 128.7, 128.5, 121.8, 116.1, 102.3, 46.9, 24.9, 23.8 HRMS (EI^+^): *m*/*z* [M + H] + calcd for C_22_H_17_N_5_O 367.1433, found 367.1435.

### 3.3. Scale up Procedure for the Synthesis of 3-[(1S)-1-Aminoethyl]-8-(2-Methoxypyrimidin-5-yl)-2-Phenyl-Isoquinolin-1-one (***3a***)

In a 2 L three-neck round-bottom flask equipped with a condenser, a mechanical stirrer, and a thermometer maintained under air atmosphere was charged with (S)-3-(1-aminoethyl)-8-chloro-2-phenylisoquinolin-1(2H)-one **1** (100.0 g, 334.7 mmol), (2-methoxypyrimidin-5-yl)boronic acid **2a** (61.8 g, 401.6 mmol), Pd (PPh_3_)_2_Cl_2_ (1.172 g, 0.5 mol%), Sphos (2.06 g, 1.5 mol%), K_2_CO_3_ (60.1 g, 435 mmol), THF (500 mL), and H_2_O (500 mL). The reaction mixture was well-stirred (120 rpm) under reflux at 65 °C for 12 h. After the biphasic reaction solution was cooled to room temperature, the organic layer was separated. The organic layer was concentrated under vacuum and diluted with CH_2_Cl_2_ (1000 mL), H_2_O (1000 mL), and conc. HCl (100 mL). The mixture was well-stirred (160 rpm) and left for 10 min. The aqueous layer was separated and washed with CH_2_Cl_2_ (500 mL). The aqueous layer was filtered and basified with NH_4_OH (200 mL). The mixture was extracted two times with CH_2_Cl_2_ (2 × 1000 mL). The combined organic layer was concentrated in vacuo. The concentrated product was recrystallized with ethyl acetate (250 mL) and added n-heptane (500 mL). The mixture was stirred at room temperature for 1hr, and the solid was collected by filtration and dried to give **3a** (118.3 g, 94.9%) as a white solid.

### 3.4. Cell Culture and Viability Assay

Three types of human cancer cell lines, such as MDA-MB-231 (metallic breast cancer cells), HeLa (cervical epithelial carcinoma cell), and HepG2 (hepatic carcinoma cell), were cultured with Dulbecco’s Modified Eagle Medium (DMEM, Welgene, Seoul, Korea) with 10% fetal bovine serum (FBS), 2 mM glutamine, and 100 units/mL antibiotics (Gibco BRL, Rockville, MD). The cells were incubated at 37 °C in a humidity atmosphere of 5% (*v*/*v*) air/CO_2_. Cells for in vitro MTT assay were inoculated into 3 × 10^5^ cells/well in a 96-cell culture palate and were stored for 24 h in a 5% CO_2_ incubator at 37 °C. Then, the cells were treated with indicated-differential concentrations of newly synthetic samples. After incubation for 24 h, 10 μL of the EZ cytox (DogenBio, Seoul, Korea) was added to each well, and the sample was further incubated for 30 min at 37 °C and 5% CO_2_ according to the manufacturer’s recommendation. The value of the cell viability was determined by measuring the formazan production with a micro-plate UV-spectrophotometer (Thermo Fisher Scientific, Waltham, MA, USA) at an absorbance of 450 nm.

## 4. Conclusions

In this study, we developed an effective method of introducing various pyrimidine groups into (*S*)-3-(1-aminoethyl)-8-chloro-2-phenylisoquinolin-1(2*H*)-one (**1**) via SMC to provide new pyrimidine-substituted isoquinoline derivatives **3**. To evaluate the activity of the compounds **3**, their cell viability (IC_50_) was measured in cancer cell lines of MDA-MB-231, HeLa, and HepG2. From the antitumor activity of compounds **3**, it was found that antitumor activity was increased when various pyrimidine rings were introduced instead of Cl at position 8 of the isoquinoline derivative **1**. This is further evidence that the pyrimidine functional group is a very good pharmacophore. In the future, we hope to identify more novel compounds with enhanced pharmacological activity using this synthetic method.

## Figures and Tables

**Table 1:** Screening of catalysts on the Suzuki–Miyaura cross-coupling of **1** with **2a** *.

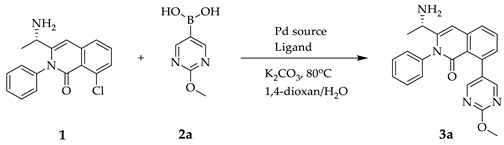				
Entry	Catalyst (Mol%)	Ligand (Mol%)	3a(%) *^b^*	1(%) *^b^*
1	Pd(PPh_3_)_4_ (5)	-	18.5	74.5
2	Pd(PPh_3_)Cl_2_ (5)	-	28.7	63.5
3	Pd(OAc)_2_ (5)	PPh_3_ (10)	19.4	73.3
4	PdCl_2_ (5)	PPh_3_ (10)	21.4	72.4
5	Pd(PPh_3_)_2_Cl_2_ (5)	PPh_3_ (10)	39.2	53.5
6	Pd(PPh_3_)_2_Cl_2_ (2.5)	PPh_3_ (5)	28.2	64.5
7	Pd(PPh_3_)_2_Cl_2_ (2.5)	P(O-tol)_3_ (5)	31.2	62.9
8	Pd(PPh_3_)_2_Cl_2_ (2.5)	P(Cy)_3_ (5)	66.2	27.4
9	Pd(PPh_3_)_2_Cl_2_ (2.5)	Dppf (5)	35.6	58.4
10	Pd(PPh_3_)_2_Cl_2_ (2.5)	Aphos (5)	76.9	17.8
11	Pd(PPh_3_)_2_Cl_2_ (2.5)	Xantphos (5)	65.6	29.2
12	Pd(PPh_3_)_2_Cl_2_ (2.5)	Xphos (5)	86.3	9.0
13	Pd(PPh_3_)_2_Cl_2_ (2.5)	Sphos (5)	96.5 *^a^*	0
14	Pd(PPh_3_)_2_Cl_2_ (2.5)	Ruphos (5)	96.4 *^a^*	0
15	Pd(PPh_3_)_2_Cl_2_ (2.5)	Davephos (5)	96.1 *^a^*	0

* Reaction conditions: 1 (1.67 mmol), 2a (2.0 mmol), K_2_CO_3_ (5 mmol), 1,4-dioxane (5 mL), H_2_O (5 mL), 12 h. *^a^* Reaction time 2 h. *^b^* The yields were determined by HPLC (area%). HPLC system using an Kromasil 100-5 C18 column (4.6 mm × 250 mm, 5 μM). The mobile phase consisted of two eluents: A: Ammonium Acetate Buffer Solution (Accurately weighed 1.54 g of ammonium acetate buffer solution is completely dissolved in 1 L of water and the pH level is adjusted to pH 4.5 with acetic acid) and eluant B: Acetonitrile: Methanol (80:20).

**Table 2:** Screening of base and solvent *.

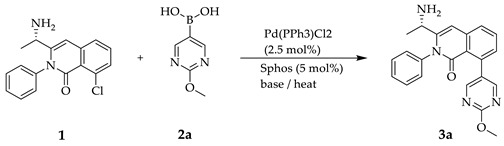					
Entry	Base	Solvent	T(℃)	3a(%) *^c^*	1(%) *^c^*
1	-	1,4-dioxane/H_2_O *^a^*	80	0	96.0
2	K_t_OBu	1,4-dioxane/H_2_O *^a^*	80	31.4	0
3	Na_2_CO_3_	1,4-dioxane/H_2_O ^*a*^	80	96.0	0
4	KOH	1,4-dioxane/H_2_O ^*a*^	80	35.4	0
5	KOAc	1,4-dioxane/H_2_O ^*a*^	80	95.1	0.1
6	Cs_2_CO_3_	1,4-dioxane/H_2_O ^*a*^	80	96.9	0.1
7	K_2_CO_3_	1,4-dioxane/H_2_O ^*a*^	80	96.9	0
8	K_2_CO_3_	THF	64 *^b^*	25.1	70.3
9	K_2_CO_3_	1,4-dioxane	104 *^b^*	9.1	67.5
10	K_2_CO_3_	EtOH	80 *^b^*	87.4	0
11	K_2_CO_3_	H_2_O	101 *^b^*	49.7	45.3
12	K_2_CO_3_	THF/H_2_O ^*a*^	65 *^b^*	99.0	0
13	K_2_CO_3_	EtOH/H_2_O ^*a*^	78 *^b^*	97.6	0

* Reaction condition: 1 (1.67 mmol), 2a (2.0 mmol), base (5 mmol), 12 h, solvent (10 mL). *^a^* Solvent (5 mL)/H_2_O(5 mL). *^b^* Reflux. *^c^* The yields were determined by HPLC (area%).

**Table 3:** Various 8-pyrimidinyl-2-phenylisoquinolin-1(2*H*)-one derivatives 3 *.

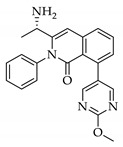	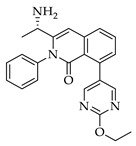	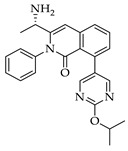	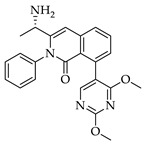
**3a** 98% ^*a*^	**3b** 97% ^*a*^	**3c** 95% ^*b*^	**3d** 45% ^*b*^
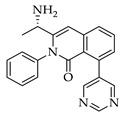	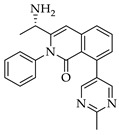	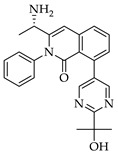	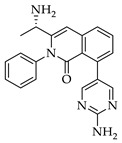
**3e** 91% ^*b*^	**3f** 95% ^*b*^	**3g** 94% ^*c*^	**3h** 61% ^*b*^
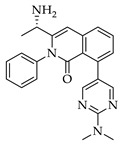	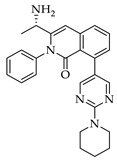	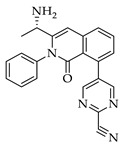	
**3i** 87% ^*a*^	**3j** 92% ^*a*^	**3k** 40% ^*d*^	

^*^ Reaction conditions: **1** (1.67 mmol), boronic acid or Pinacol boronate (2.0 mmol), Pd(PPh_3_)_2_Cl_2_ (0.5~5 mol%), Sphos (1.5–15 mol%), K_2_CO_3_ (5.0 mmol), 12 hr, THF (5 mL)/H_2_O (5 mL), Isolated yield. *^a^* boronic acid (2.0 mmol), Pd(PPh_3_)_2_Cl_2_ (0.5 mol%), Sphos (1.5 mol%). *^b^* boronic acid (2.0 mmol), Pd(PPh_3_)_2_Cl_2_ (5 mol%), Sphos (15 mol%) *^c^* Pinacol boronate (2.0 mmol), Pd(PPh_3_)_2_Cl_2_ (1.0 mol%), Sphos (3 mol%). *^d^* Pinacol boronate (2.0 mmol), Pd(PPh_3_)_2_Cl_2_ (5 mol%), Sphos (15 mol%).

**Table 4:** Selected IC_50_ values of **3** against different cancer cell lines.

Entry	Compound	MDA-MB231 IC_50_ (μM) *^a^*	HeLa IC_50_ (μM) *^a^*	HepG2 IC_50_ (μM) *^a^*
1	**1**	ND *^b^*	ND *^b^*	ND *^b^*
2	**3a**	ND *^b^*	5.07 ± 0.13	2.20 ± 0.26
3	**3b**	2.28 ± 0.10	1.55 ± 0.15	2.01 ± 0.05
4	**3c**	2.83 ± 0.10	1.86 ± 0.12	1.79 ± 0.13
5	**3d**	ND *^b^*	2.07 ± 0.46	4.85 ± 0.21
6	**3e**	ND *^b^*	2.12 ± 0.17	5.12 ± 0.23
7	**3f**	ND *^b^*	5.38 ± 0.22	5.22 ± 0.23
8	**3g**	5.25 ± 0.09	2.43 ± 0.25	5.27 ± 0.39
9	**3h**	2.72 ± 0.24	1.94 ± 0.11	2.93 ± 0.10
10	**3i**	2.29 ± 0.11	1.42 ± 0.19	3.08 ± 0.20
11	**3j**	1.62 ± 0.12	1.11 ± 0.70	3.87 ± 1.58
12	**3k**	1.18 ± 0.08	1.99 ± 0.28	1.57 ± 0.17

*^a^* MTT assay. *^b^* ND: Not determined. At the 10 μM, less than 50% cell death was observed. The viability test was performed in triplicate and repeated at least three times. The cell viability represents the mean ± SD from three independent experiments.

## Data Availability

Data are contained within the article.

## Supplementary Materials

The following are available online at https://www.mdpi.com/article/10.3390/ph15010064/s1. Analytical data for synthesized compounds. ^1^H NMR, ^13^C NMR for compounds **3a**–**3k**: Figure S1–S22; Cell toxicity data of compounds 1, **3a**–**3k** for MDA-MB 231, HeLa, HepG2: Figure S23–S34.
