# Supplementary material for: Synthesis of Novel (S)-3-(1-Aminoethyl)-8-pyrimidinyl-2-phenylisoquinolin-1(2H)-ones by Suzuki–Miyaura Coupling and Their Cell Toxicity Activities
Source: Pharmaceuticals (Basel). 2022 Jan 4;15(1):64. doi: 10.3390/ph15010064 (PMC8779526; doi:10.3390/ph15010064)
Supplement: Supplementary file 1 [file pharmaceuticals-15-00064-s001.zip › pharmaceuticals-1515907-supplementary.pdf]

Synthesis of novel (*S*)-3-(1-aminoethyl)-8-pyrimidinyl-2-phenylisoquinolin-1(2*H*)-ones by Suzuki-Miyaura coupling and their cell toxicity activities

Ok Kyoung Choi[1,2], Yong Ho Sun[2], Hyemi Lee[2], Joon Kwang Lee[2], Taehoon Lee[1,*], Hakwon Kim[1,*]

[1] Department of Applied Chemistry and Global Center for Pharmaceutical Ingredient Materials, Kyung Hee University, Yongin 17410, Republic of Korea
[2] Process Resarch, Boryung R&D Institute, Ansan 15425, Republic of Korea
* Correspondence: hwkim@khu.ac.kr (H.K.); thlee@khu.ac.kr (T. L.); Tel.: +82-31-201-2459 (H.K.); +82-31-201-5173 (T. L.)

Table of Contents:

^{1}H and ^{13}C NMR Spectra …………………..……………………………… page 2 - 12

Cell toxicity …..……………………..……………………………… page 13 - 17

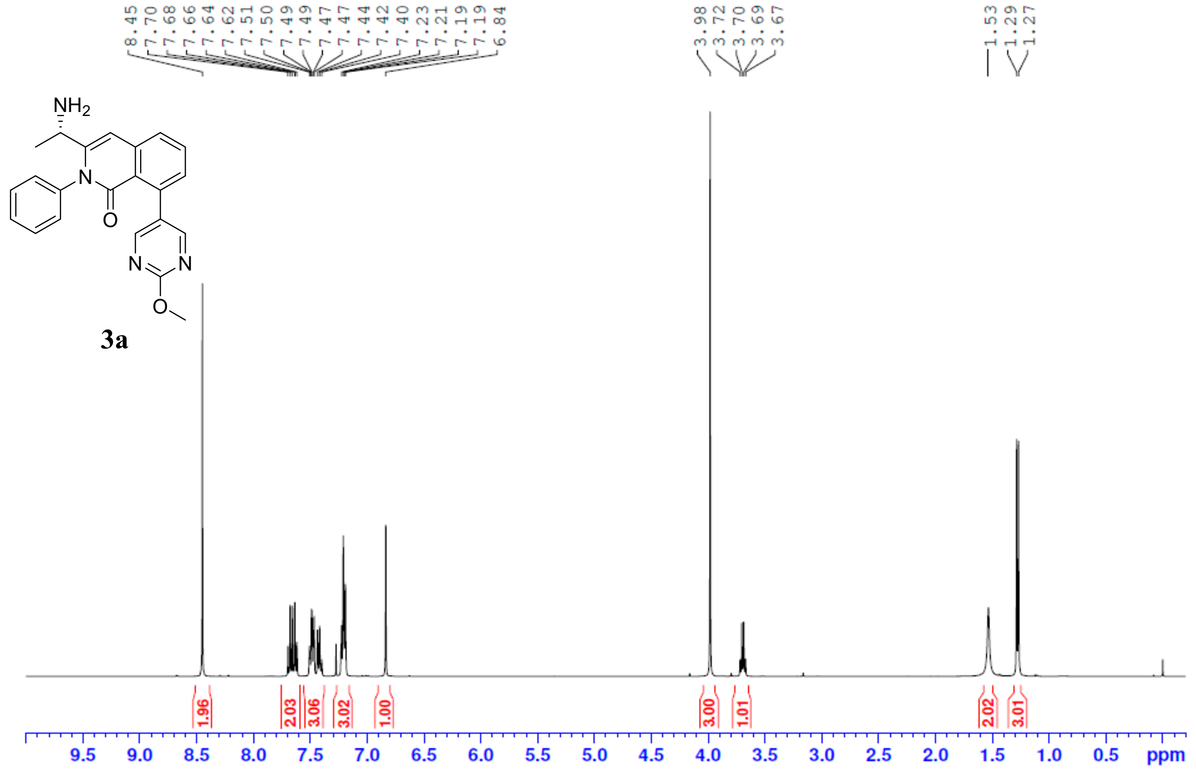

Figure S1. ^{1}H NMR Spectrum of 3-[(1*S*)-1-aminoethyl]-8-(2-methoxypyrimidin-5-yl)-2-phenyl-isoquinolin-1-one (**3a**)

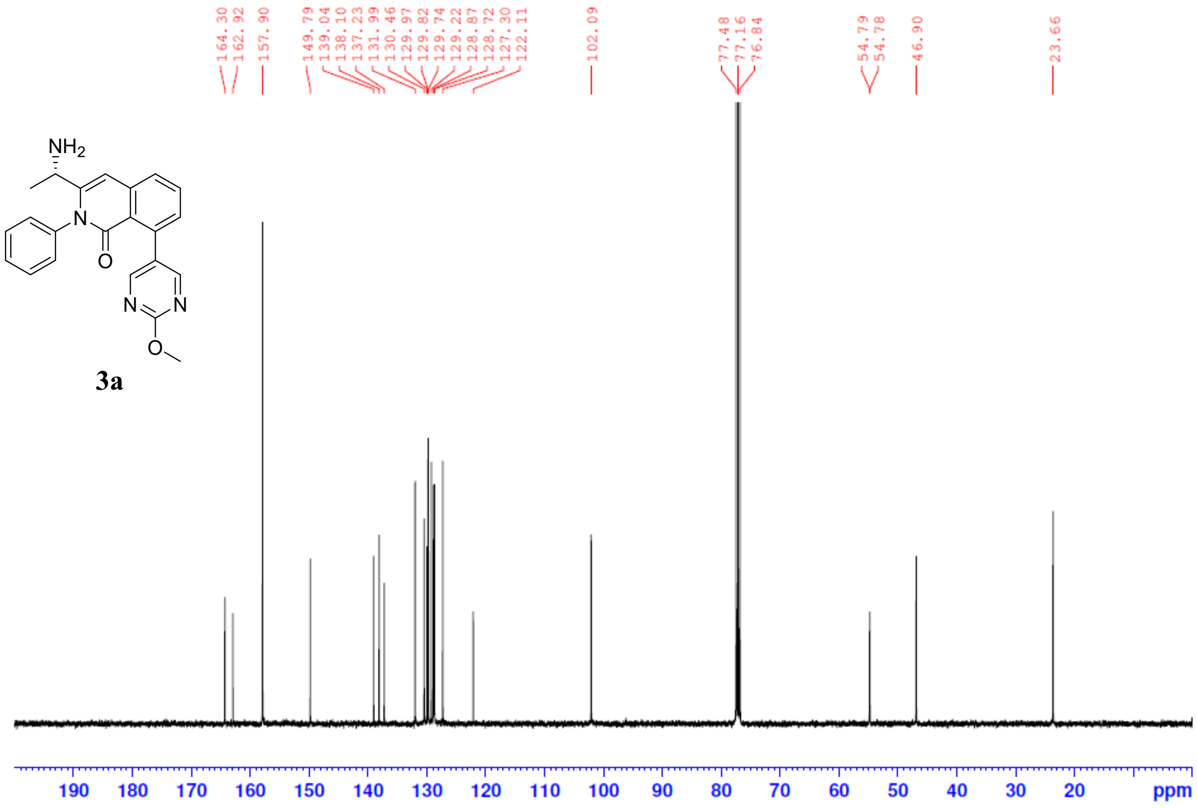

Figure S2. ^{13}C NMR Spectrum of 3-[(1*S*)-1-aminoethyl]-8-(2-methoxypyrimidin-5-yl)-2-phenyl-isoquinolin-1-one (**3a**)

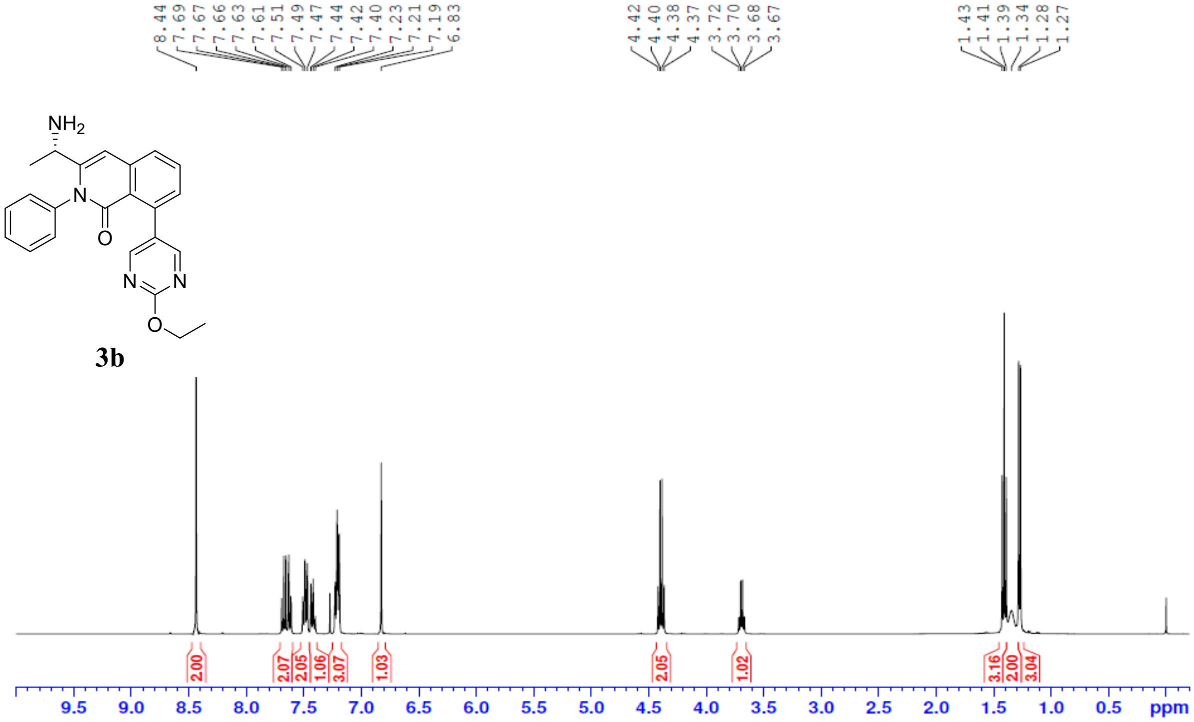

Figure S3. ^{1}H NMR Spectrum of 3-[(1*S*)-1-aminoethyl]-8-(2-ethoxypyrimidin-5-yl)-2-phenyl-isoquinolin-1-one (**3b**)

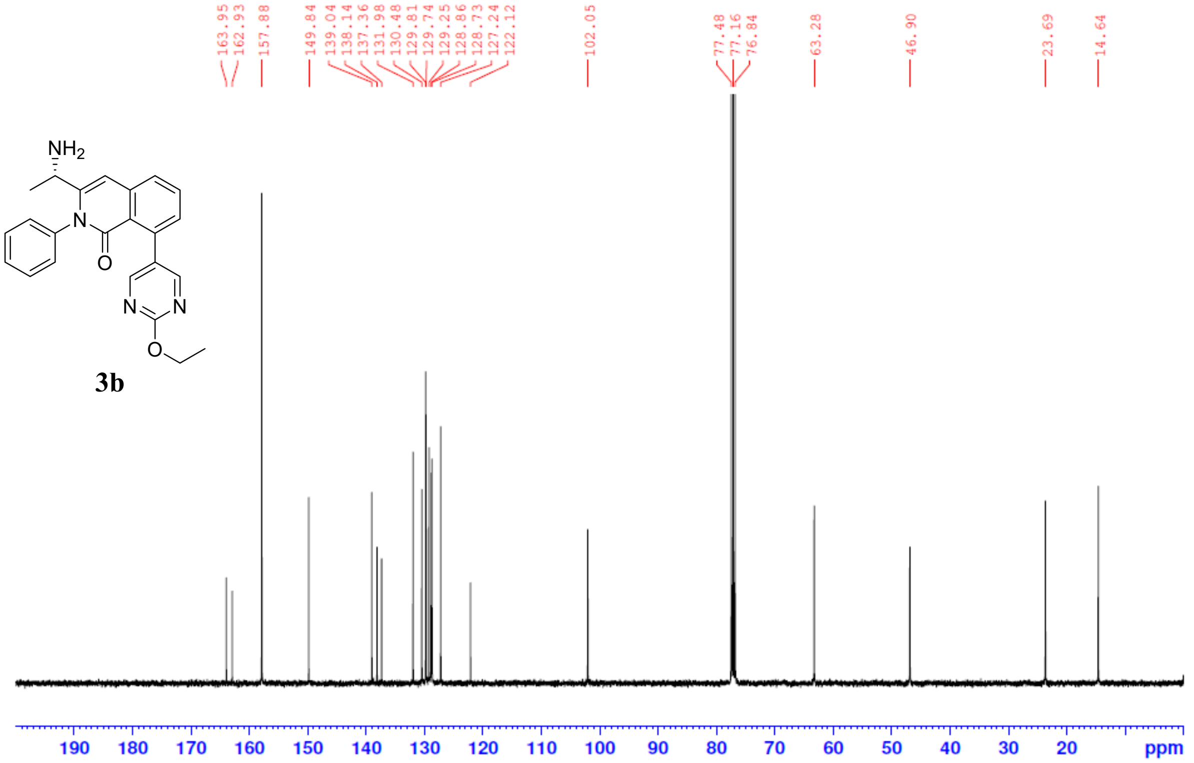

Figure S4. ^{13}C NMR Spectrum of 3-[(1*S*)-1-aminoethyl]-8-(2-ethoxypyrimidin-5-yl)-2-phenyl-isoquinolin-1-one (**3b**)

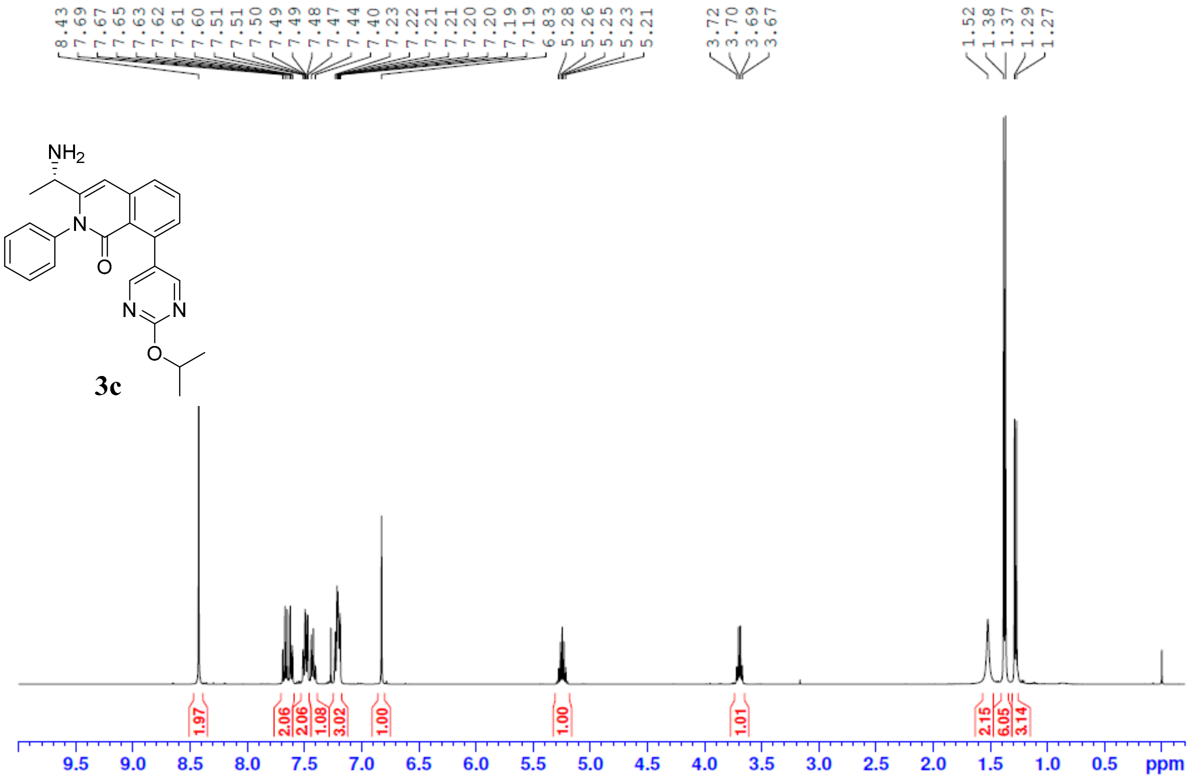

Figure S5. ^{1}H NMR Spectrum of 3-[(1*S*)-1-aminoethyl]-8-(2-isopropoxypyrimidin-5-yl)-2-phenyl-isoquinolin-1-one (**3c**)

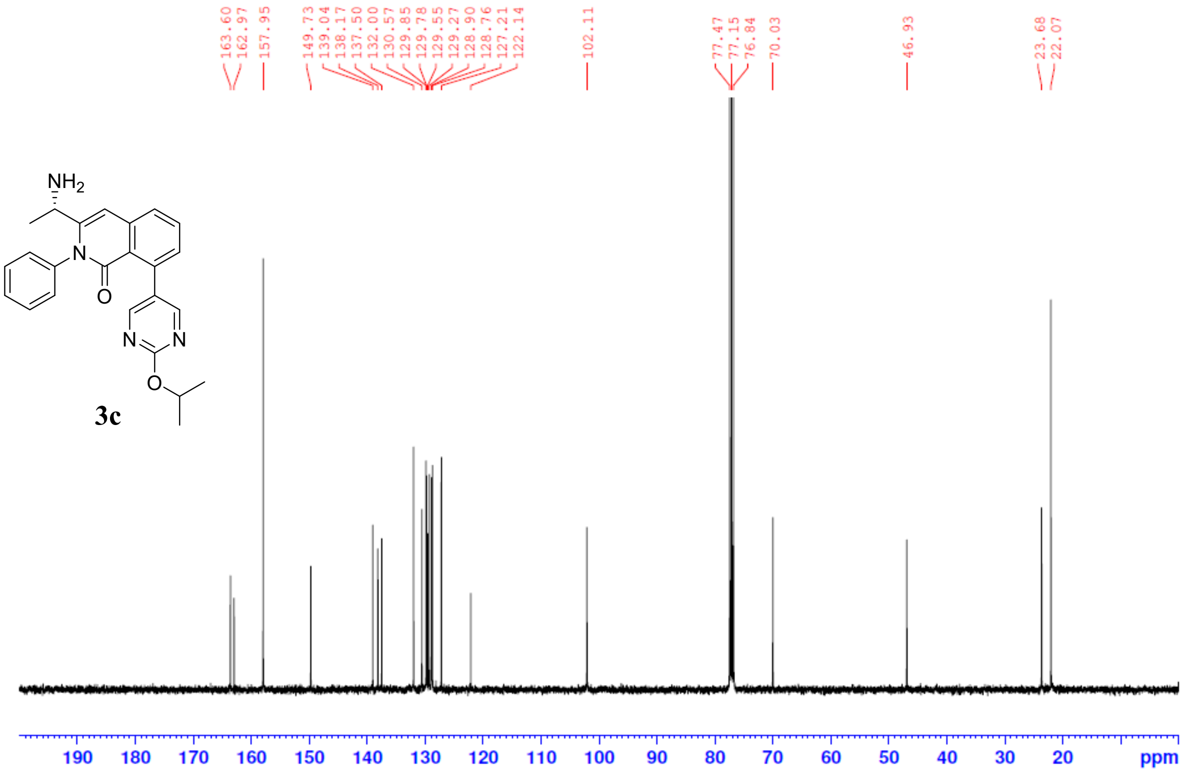

Figure S6. ^{13}C NMR Spectrum of 3-[(1*S*)-1-aminoethyl]-8-(2-isopropoxypyrimidin-5-yl)-2-phenyl-isoquinolin-1-one (**3c**)

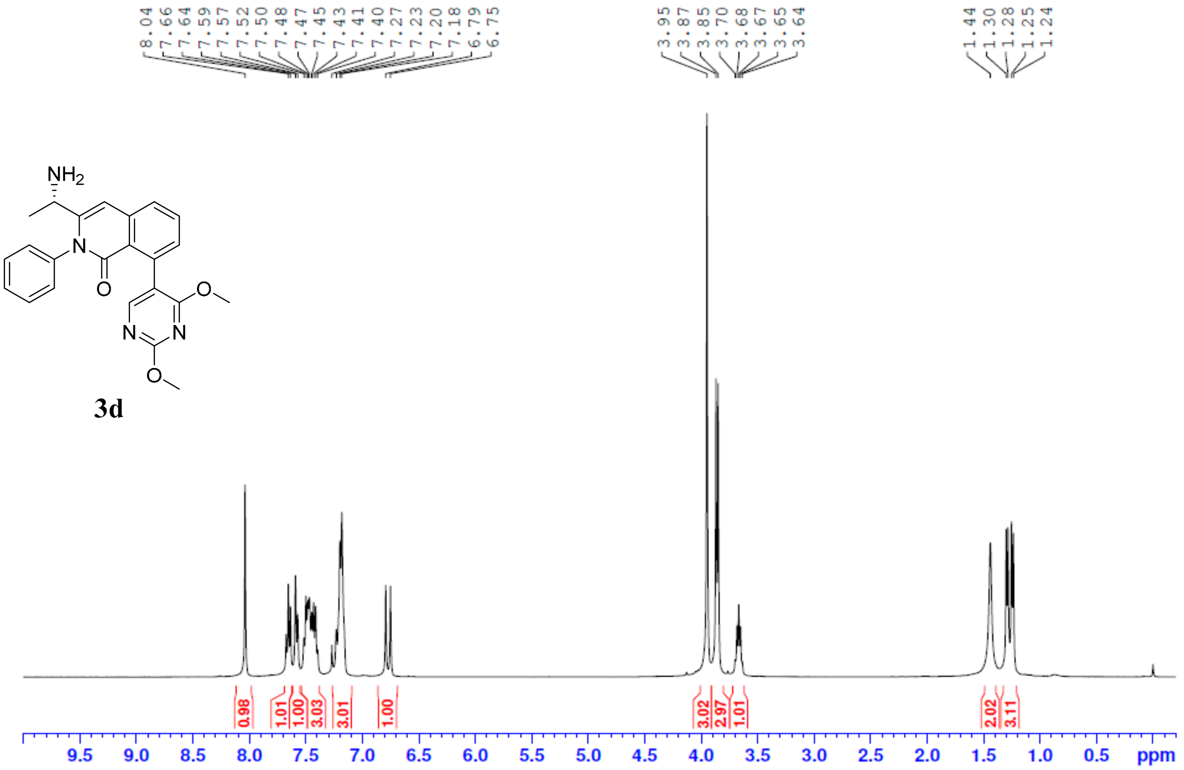

3d

Figure S7. ^{1}H NMR Spectrum of 3-[(1*S*)-1-aminoethyl]-8-(2,4-dimethoxypyrimidin-5-yl)-2-phenyl-isoquinolin-1-one (**3d**)

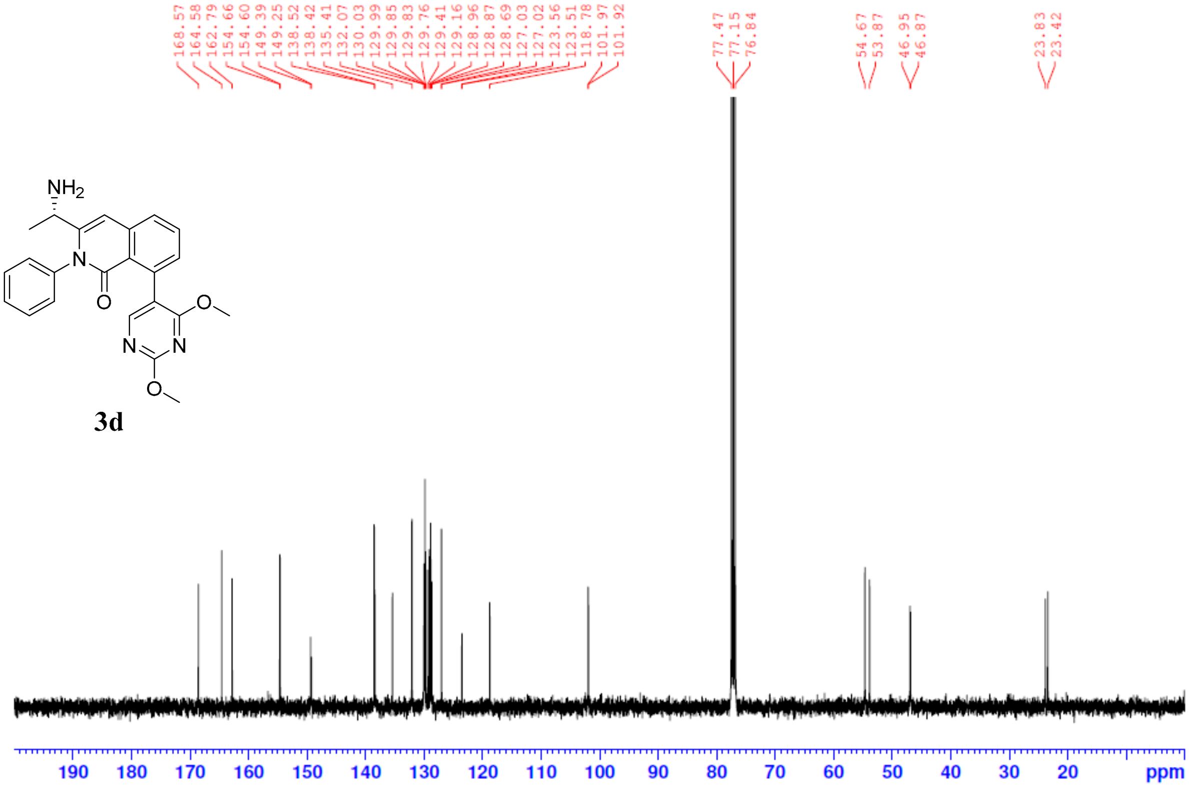

3d

Figure S8. ^{13}C NMR Spectrum of 3-[(1*S*)-1-aminoethyl]-8-(2,4-dimethoxypyrimidin-5-yl)-2-phenyl-isoquinolin-1-one (**3d**)

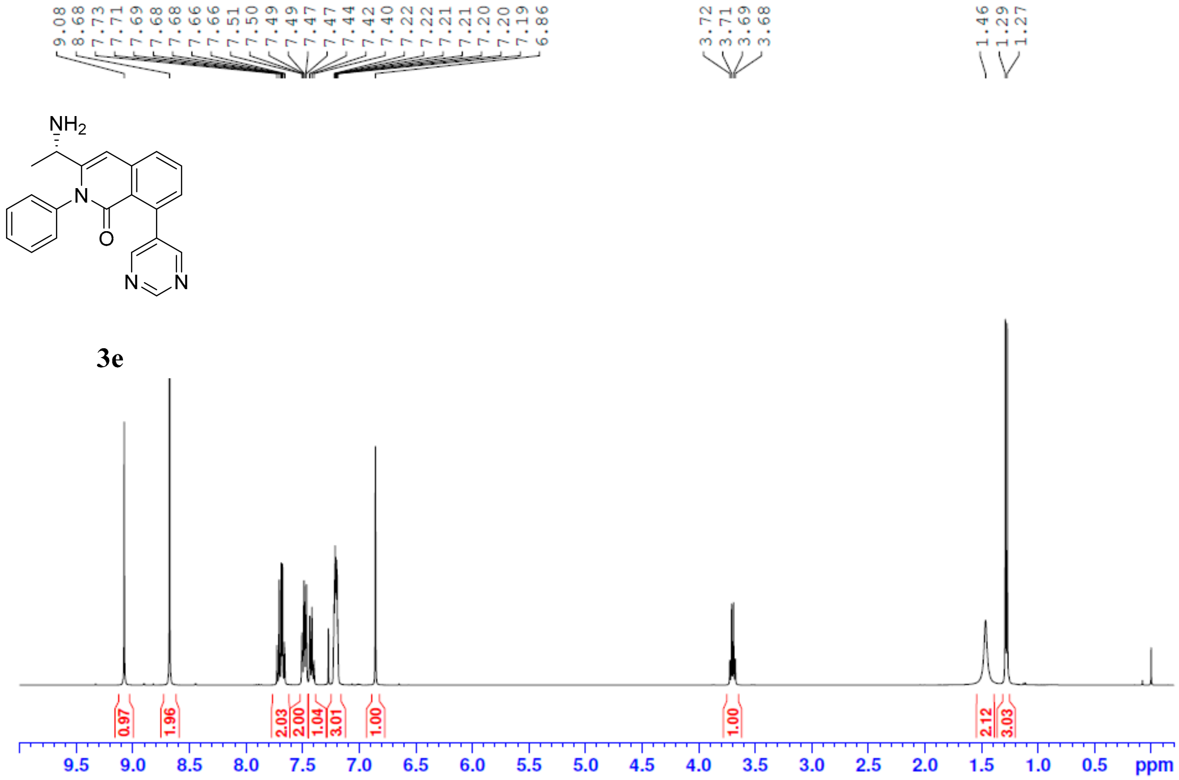

Figure S9. ^{1}H NMR Spectrum of 3-[(1*S*)-1-aminoethyl]-2-phenyl-8-pyrimidin-5-yl-isoquinolin-1-one (**3e**)

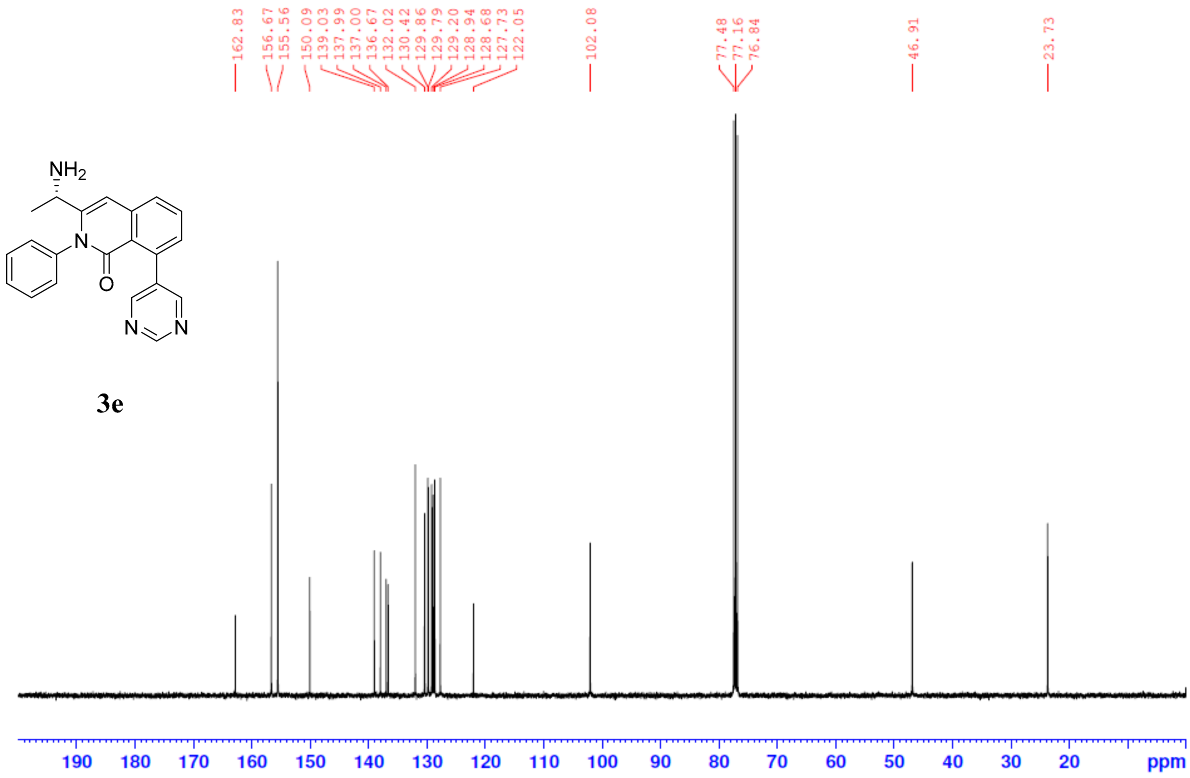

Figure S10. ^{13}C NMR Spectrum of 3-[(1*S*)-1-aminoethyl]-2-phenyl-8-pyrimidin-5-yl-isoquinolin-1-one (**3e**)

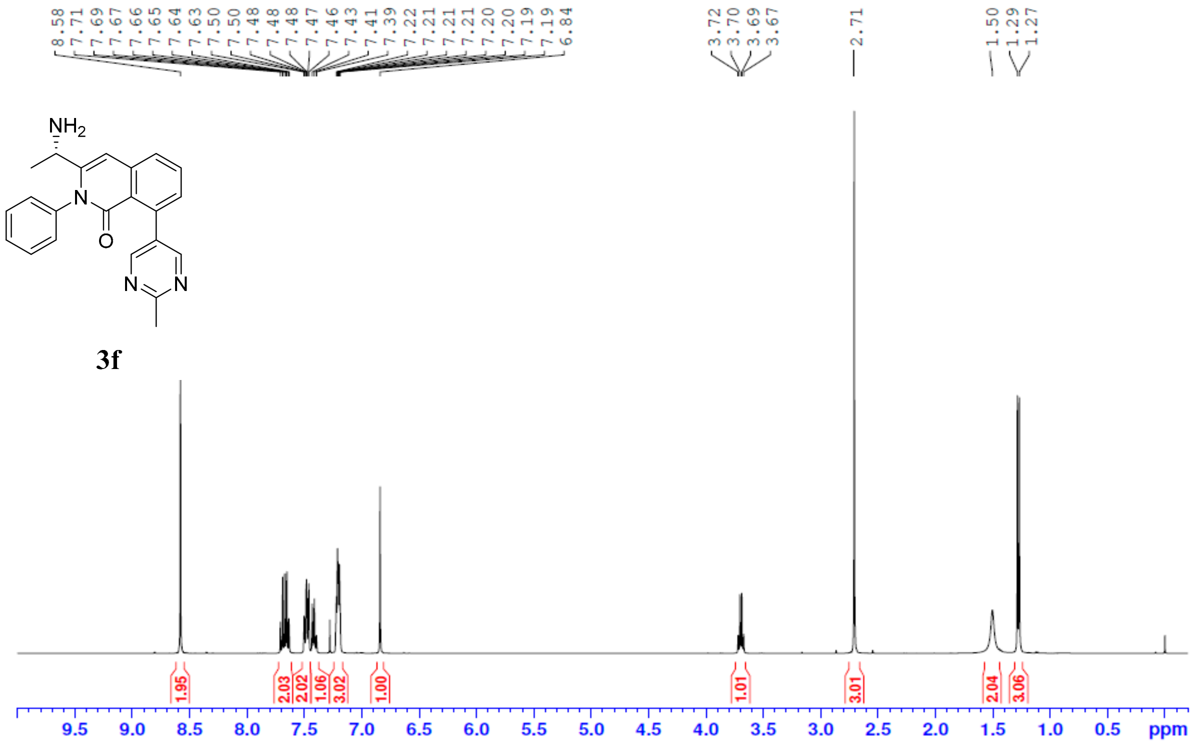

Figure S11. ¹H NMR Spectrum of 3-[(1*S*)-1-aminoethyl]-8-(2-methylpyrimidin-5-yl)-2-phenyl-isoquinolin-1-one (**3f**)

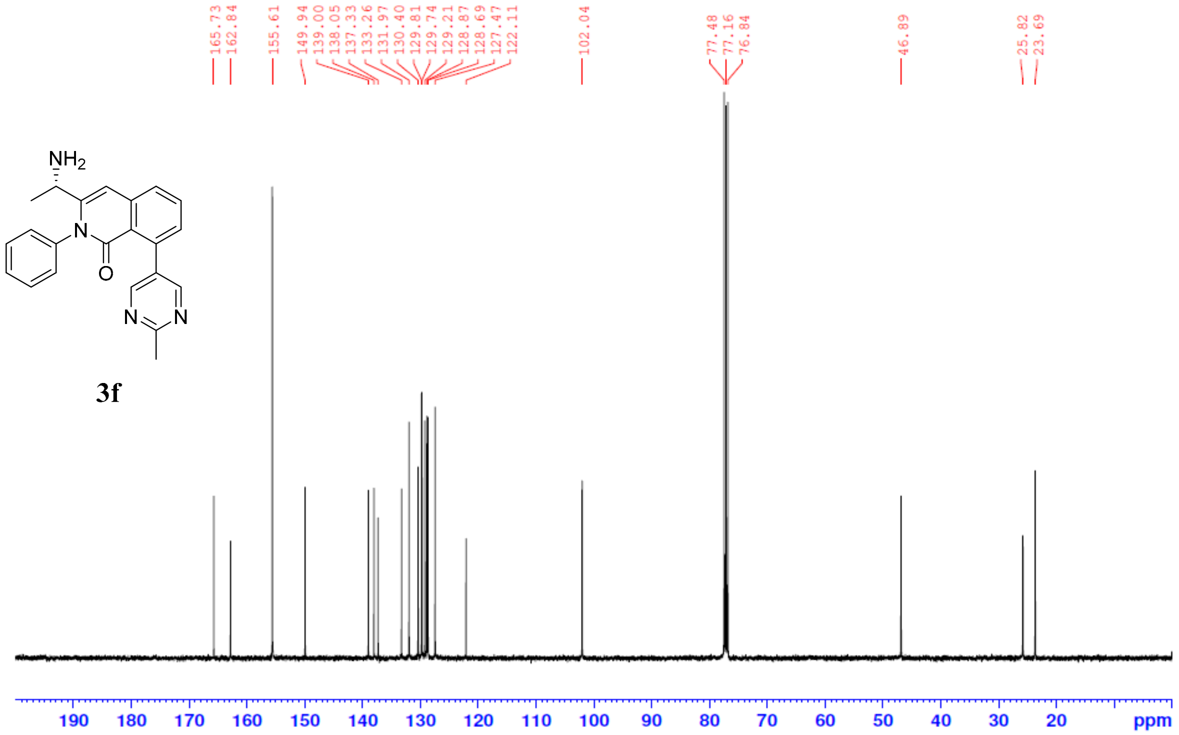

Figure S12. ¹³C NMR Spectrum of 3-[(1*S*)-1-aminoethyl]-8-(2-methylpyrimidin-5-yl)-2-phenyl-isoquinolin-1-one (**3f**)

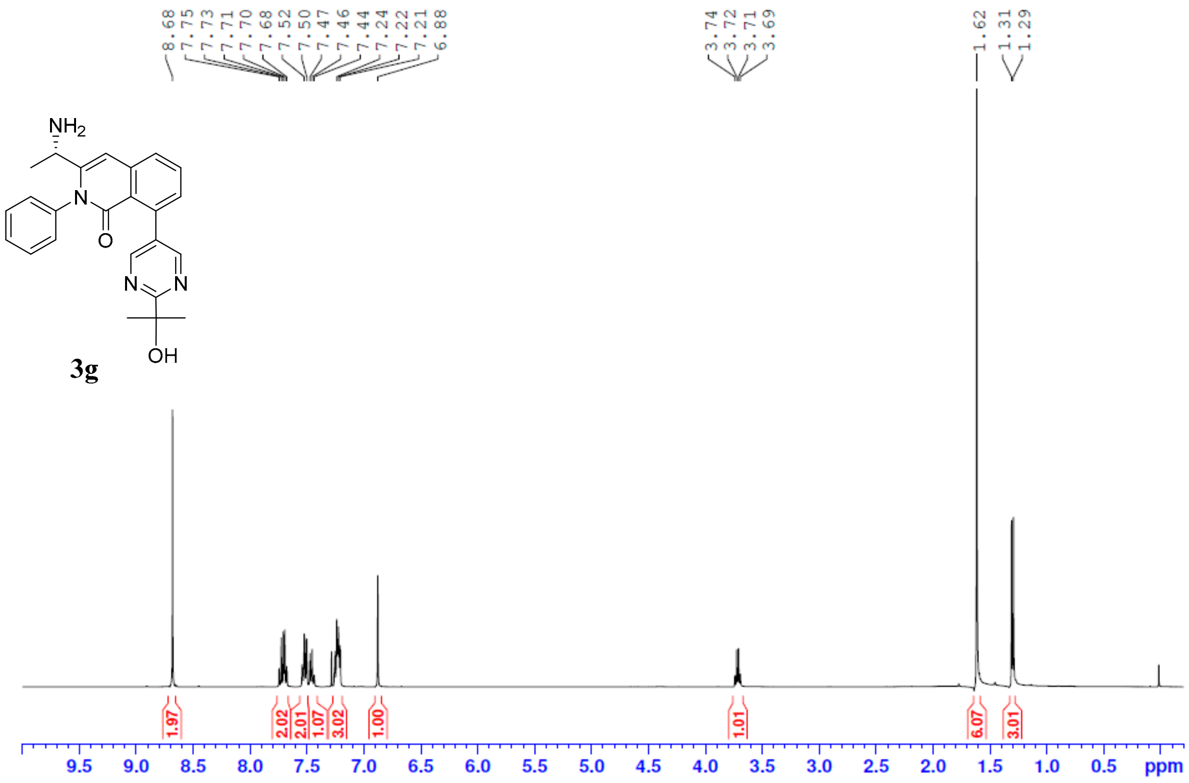

Figure S13. ^{1}H NMR Spectrum of 3-[(1*S*)-1-aminoethyl]-8-[2-(1-hydroxy-1-methyl-ethyl)pyrimidin-5-yl]-2-phenyl-isoquinolin-1-one (**3g**)

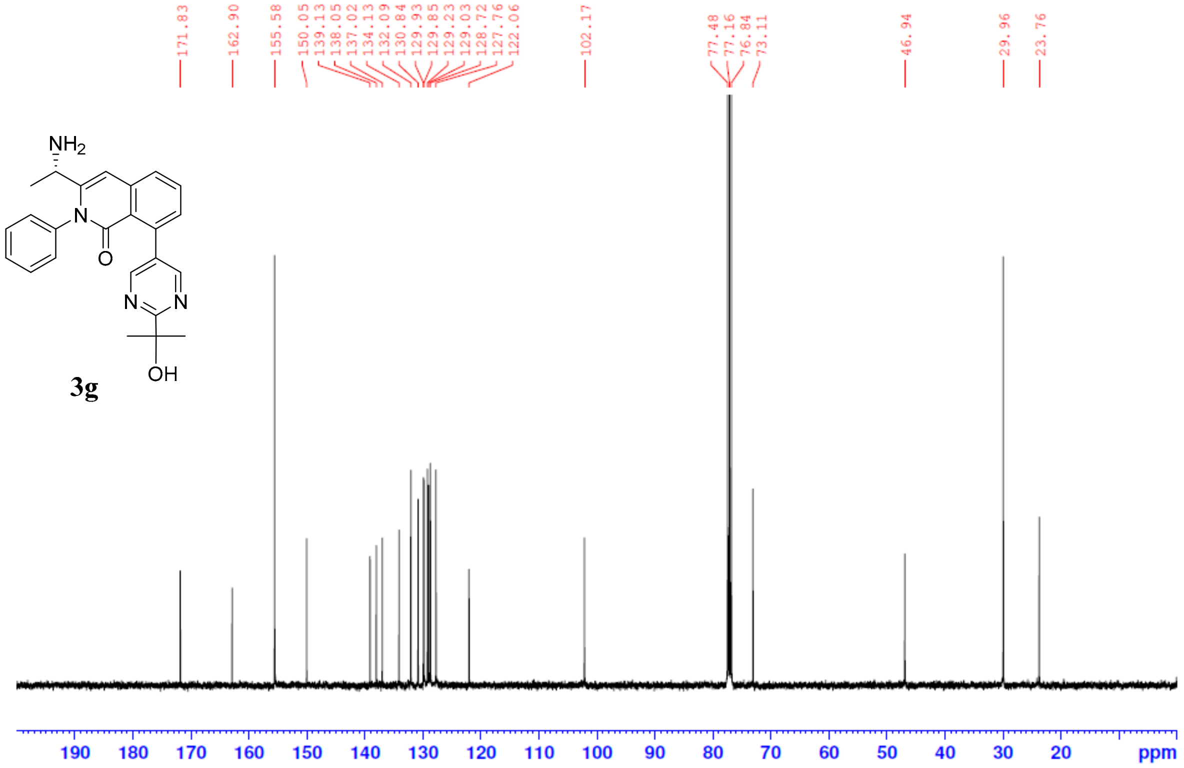

Figure S14. ^{13}C NMR Spectrum of 3-[(1*S*)-1-aminoethyl]-8-[2-(1-hydroxy-1-methyl-ethyl)pyrimidin-5-yl]-2-phenyl-isoquinolin-1-one (**3g**)

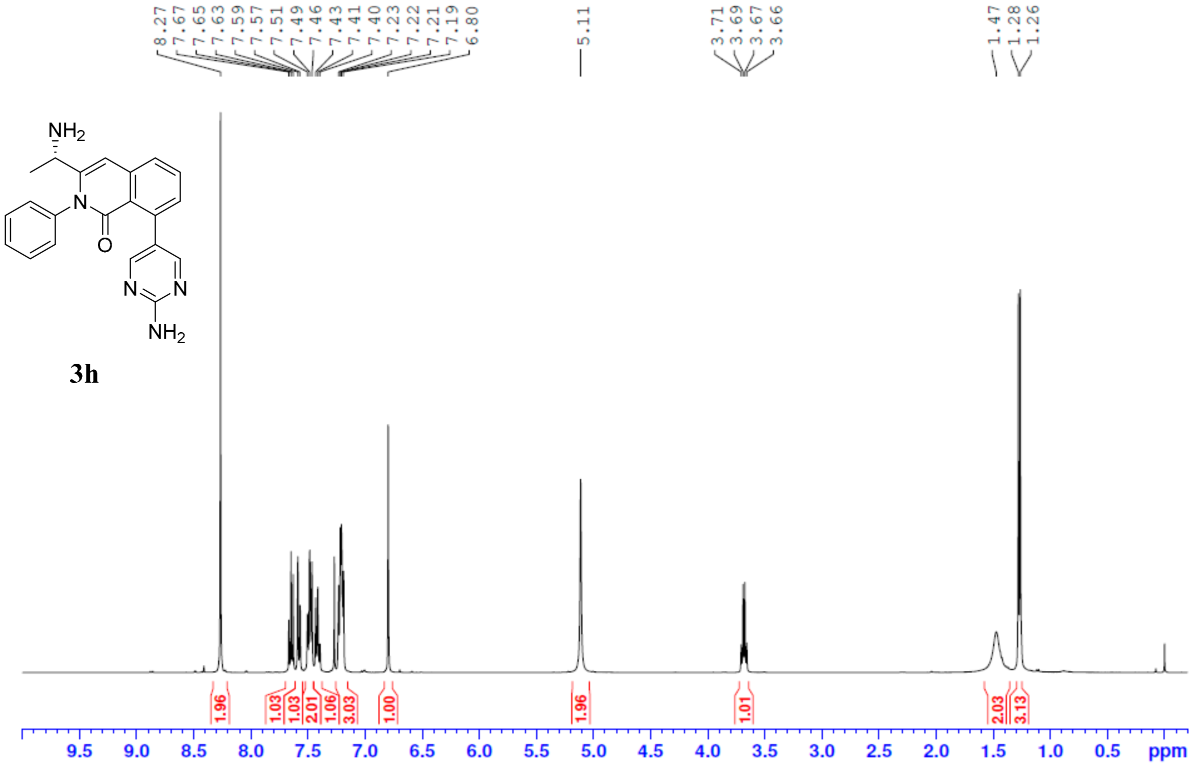

Figure S15. ^{1}H NMR Spectrum of 3-[(1*S*)-1-aminoethyl]-8-(2-aminopyrimidin-5-yl)-2-phenyl-isoquinolin-1-one (**3h**)

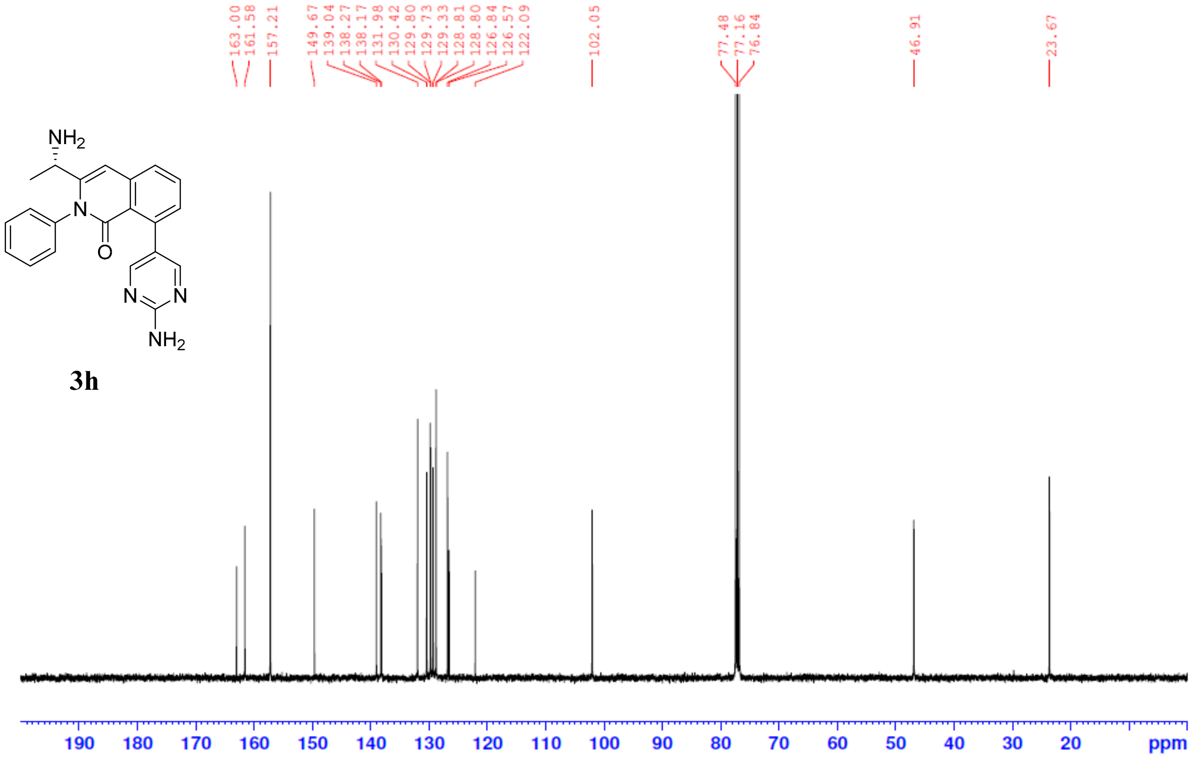

Figure S16. ^{13}C NMR Spectrum of 3-[(1*S*)-1-aminoethyl]-8-(2-aminopyrimidin-5-yl)-2-phenyl-isoquinolin-1-one (**3h**)

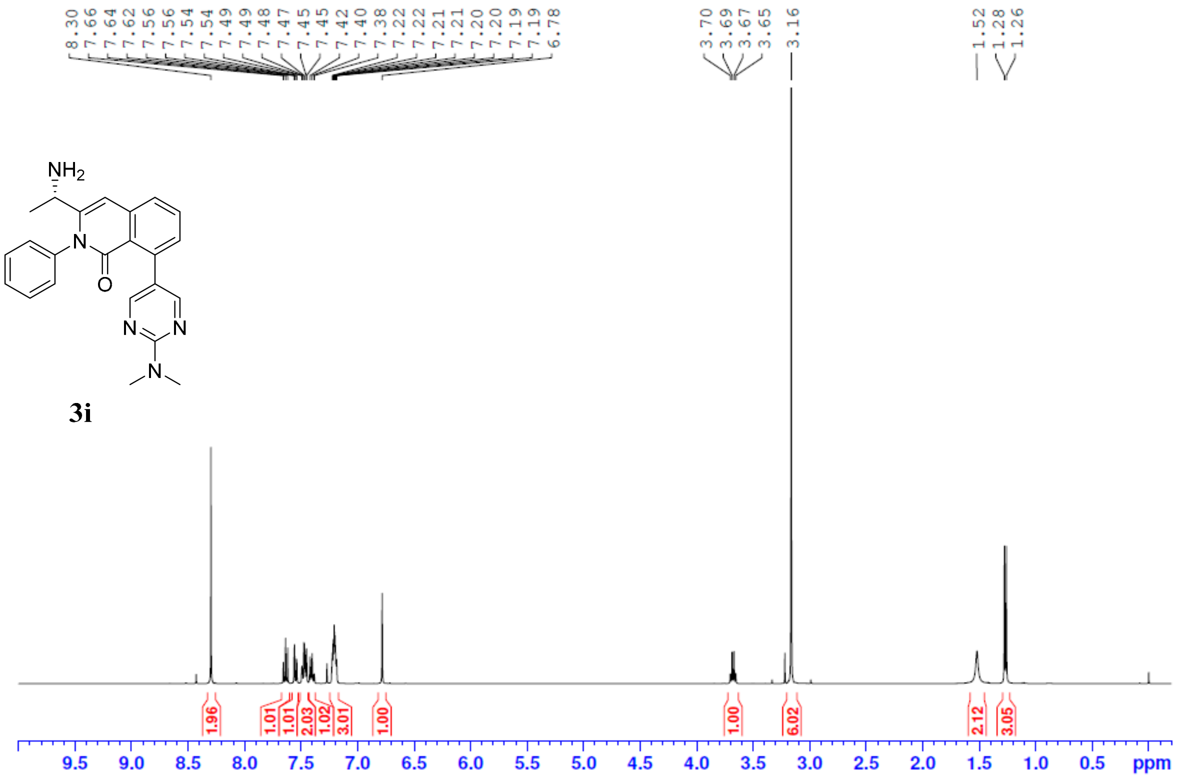

Figure S17. ^{1}H NMR Spectrum of 3-[(1*S*)-1-aminoethyl]-8-[2-(dimethylamino)pyrimidin-5-yl]-2-phenyl-isoquinolin-1-one (**3i**)

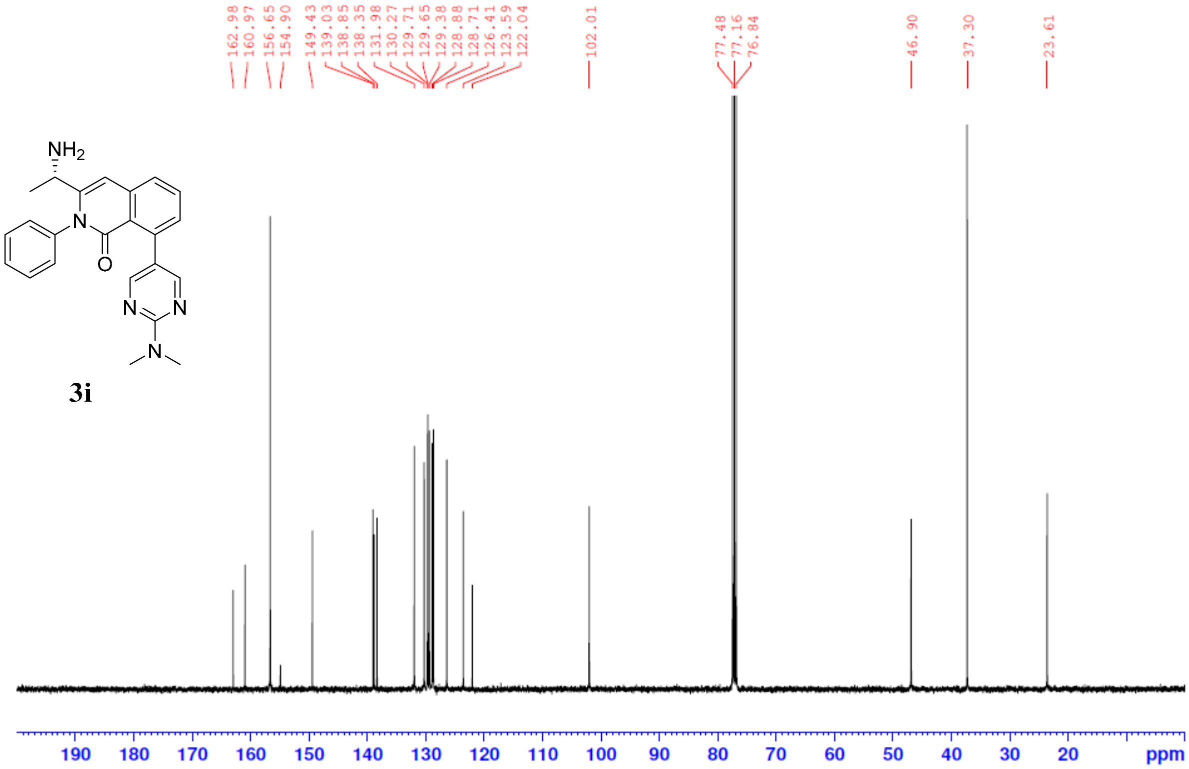

Figure S18. ^{13}C NMR Spectrum of 3-[(1*S*)-1-aminoethyl]-8-[2-(dimethylamino)pyrimidin-5-yl]-2-phenyl-isoquinolin-1-one (**3i**)

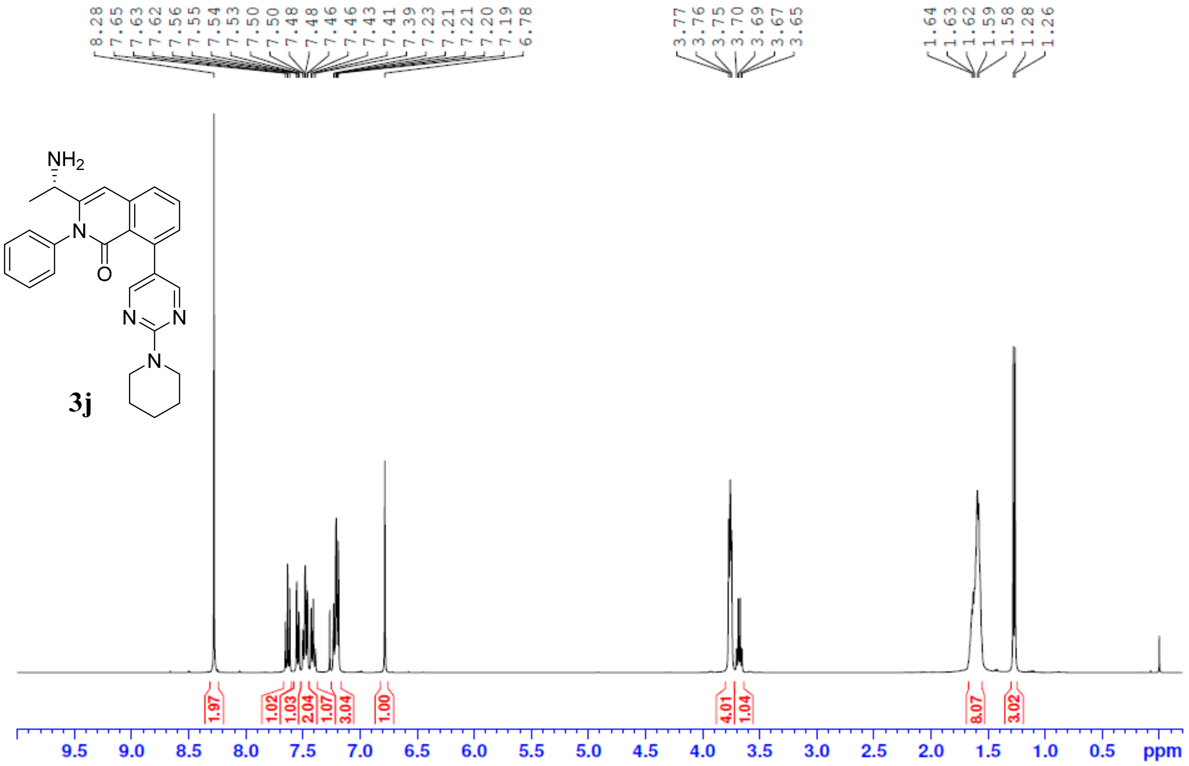

Figure S19. ^{1}H NMR Spectrum of 3-[(1*S*)-1-aminoethyl]-2-phenyl-8-[2-(1-piperidyl)pyrimidin-5-yl]isoquinolin-1-one (**3j**)

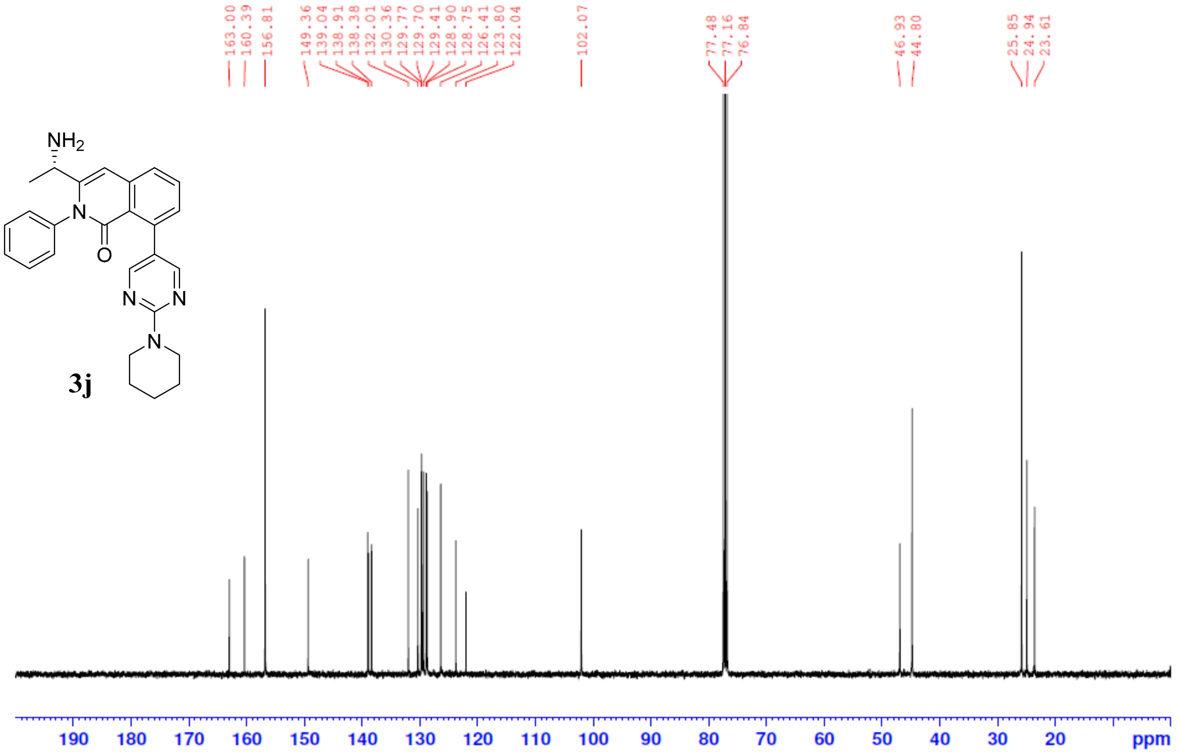

Figure S20. ^{13}C NMR Spectrum of 3-[(1*S*)-1-aminoethyl]-2-phenyl-8-[2-(1-piperidyl)pyrimidin-5-yl]isoquinolin-1-one (**3j**)

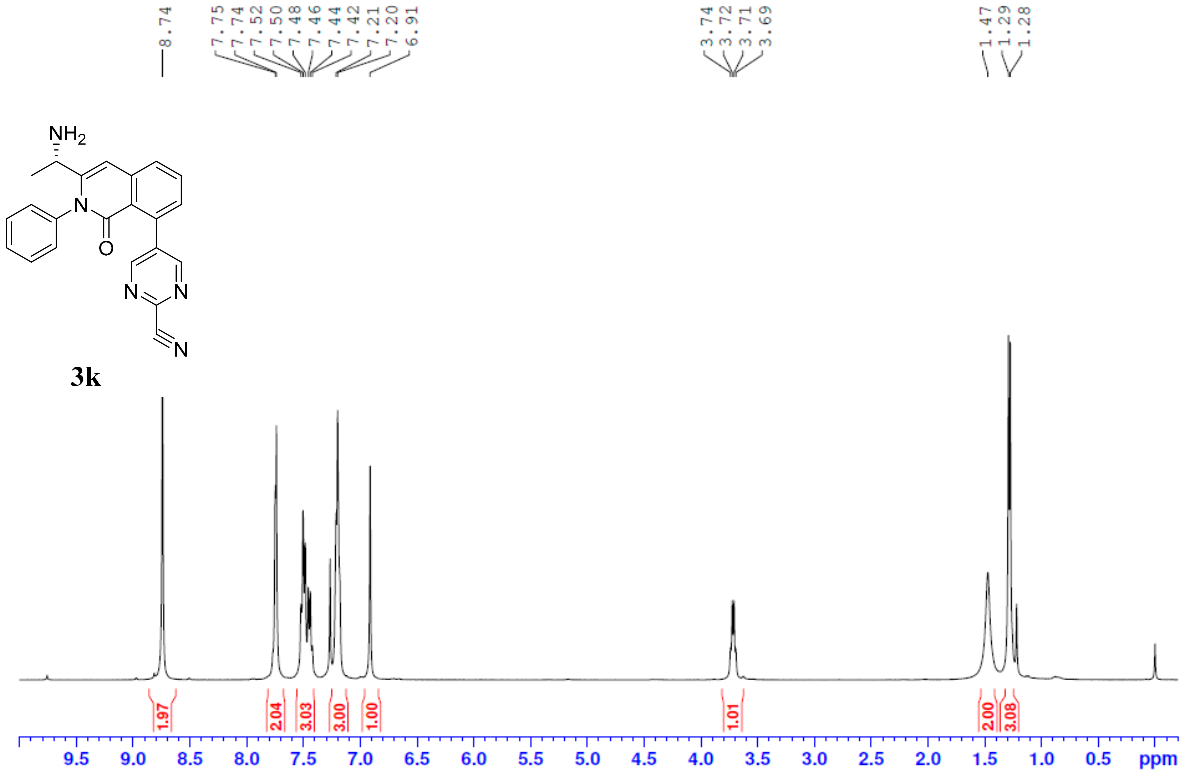

Figure S21. ^{1}H NMR Spectrum of 5-[3-[(1*S*)-1-aminoethyl]-1-oxo-2-phenyl-8-isoquinolyl]pyrimidine-2-carbonitrile (**3k**)

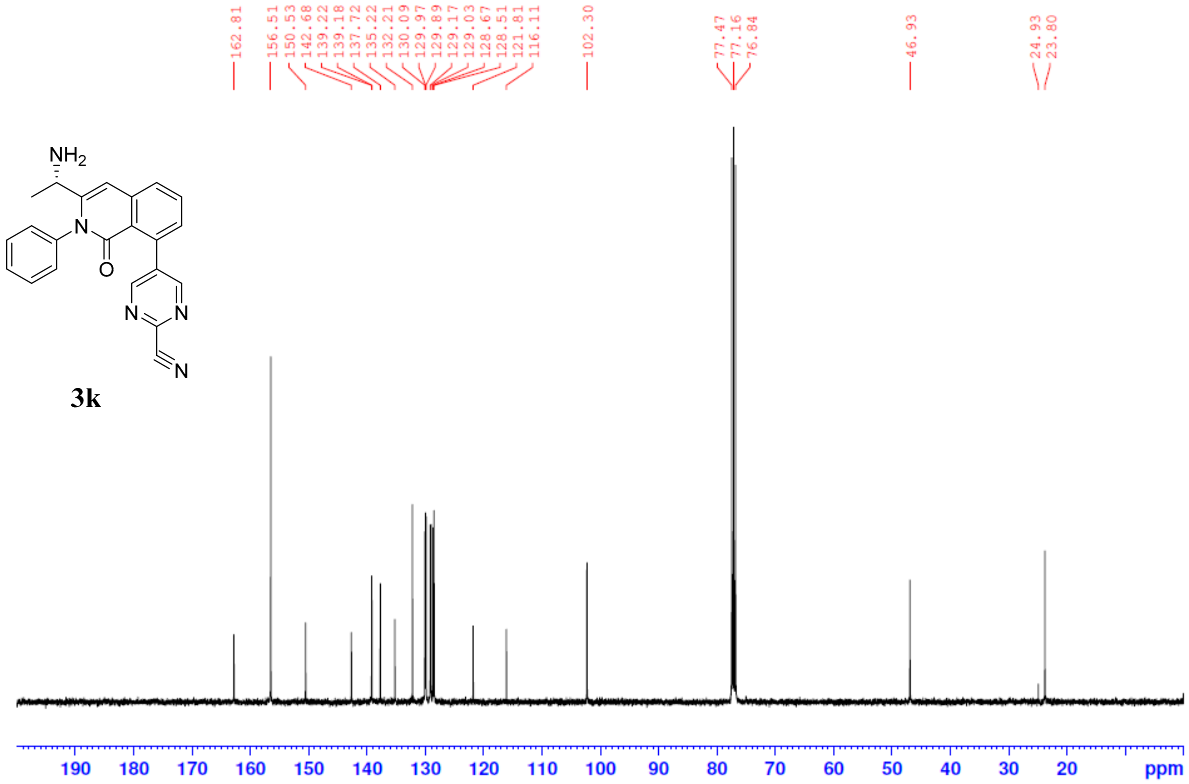

Figure S22. ^{13}C NMR Spectrum of 5-[3-[(1*S*)-1-aminoethyl]-1-oxo-2-phenyl-8-isoquinolyl]pyrimidine-2-carbonitrile (**3k**)

MTT cytotoxicity assay

The cell viability of the cultured cells was determined by measuring the reduction of 3-(4,5-dimethylthiazol-2-yl)-2,5-diphenyltetrazolium bromide (MTT) to formazan. Briefly, cells were seeded and treated with different concentrations of the synthesized compounds. After incubation with LPS (100 ng/mL) for 24 h, 0.5 mg/mL of MTT solution was added to each well. After incubation for 2 h at 37 °C and 5% CO_2, the supernatants were removed and the formed formazan crystals in the viable cells were dissolved in dimethyl sulfoxide (DMSO). The absorbance at 550 nm was determined using a microplate reader (Molecular device, USA).

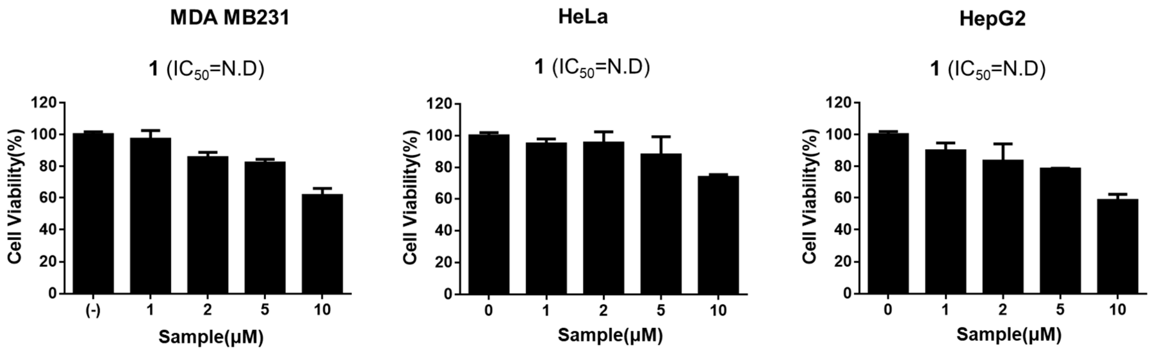

Figure S23. Cell toxicity of (*S*)-3-(1-aminoethyl)-8-chloro-2-phenylisoquinolin-1(2H)-one (**1**) for MDA-MB 231, HeLa, HepG2.

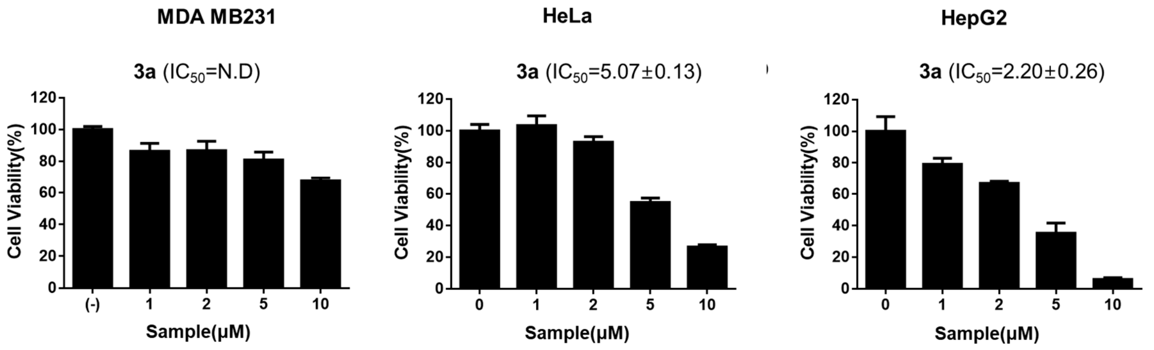

Figure S24. Cell toxicity of 3-[(1*S*)-1-aminoethyl]-8-(2-methoxypyrimidin-5-yl)-2-phenyl-isoquinolin-1-one (**3a**) for MDA-MB 231, HeLa, HepG2.

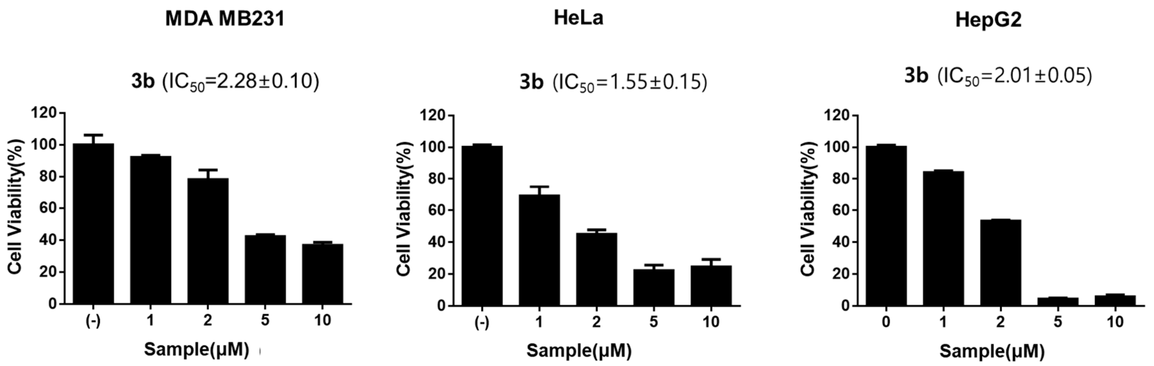

Figure S25. Cell toxicity of 3-[(1*S*)-1-aminoethyl]-8-(2-ethoxypyrimidin-5-yl)-2-phenyl-isoquinolin-1-one (**3b**) for MDA-MB 231, HeLa, HepG2.

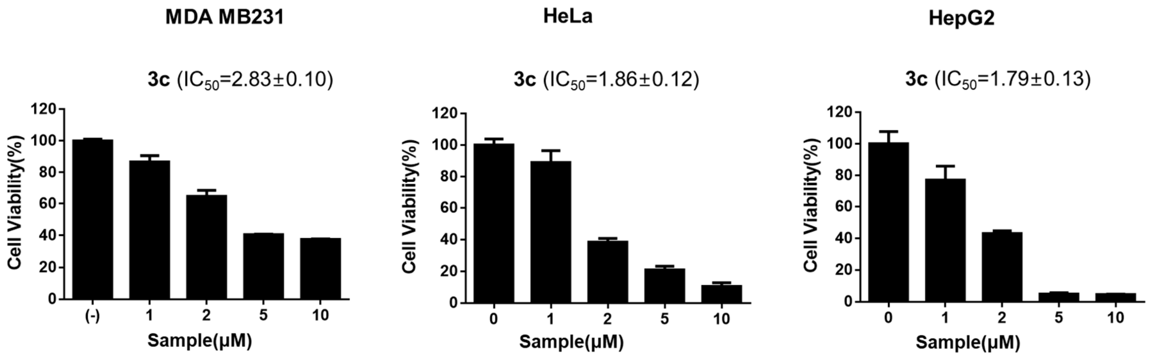

Figure S26. Cell toxicity of 3-[(1*S*)-1-aminoethyl]-8-(2-isopropoxypyrimidin-5-yl)-2-phenyl-isoquinolin-1-one (**3c**) for MDA-MB 231, HeLa, HepG2.

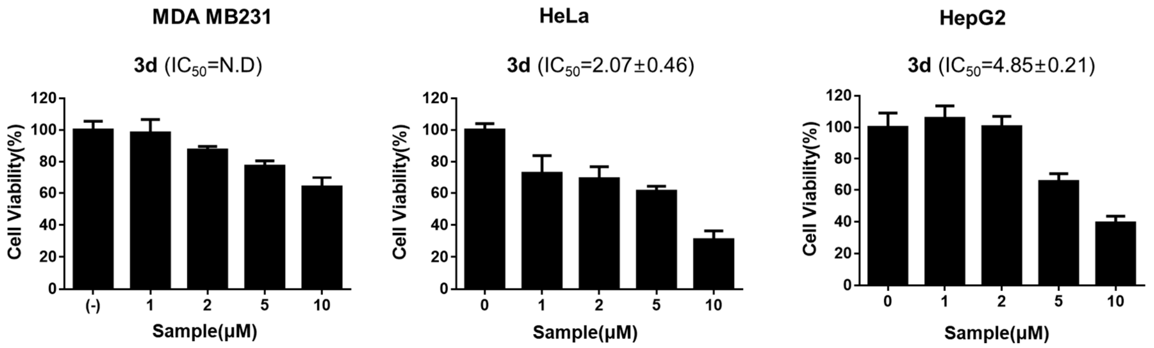

Figure S27. Cell toxicity of 3-[(1*S*)-1-aminoethyl]-8-(2,4-dimethoxypyrimidin-5-yl)-2-phenyl-isoquinolin-1-one (**3d**) for MDA-MB 231, HeLa, HepG2.

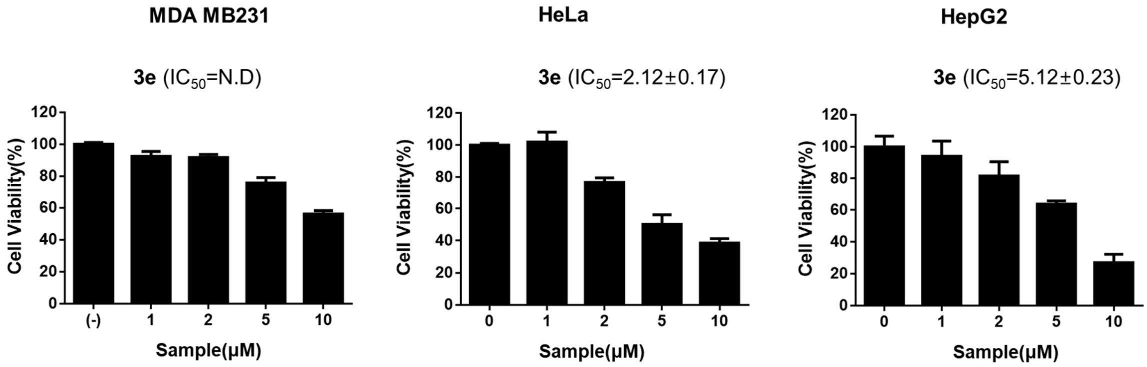

Figure S28. Cell toxicity of 3-[(1*S*)-1-aminoethyl]-2-phenyl-8-pyrimidin-5-yl-isoquinolin-1-one (**3e**) for MDA-MB 231, HeLa, HepG2.

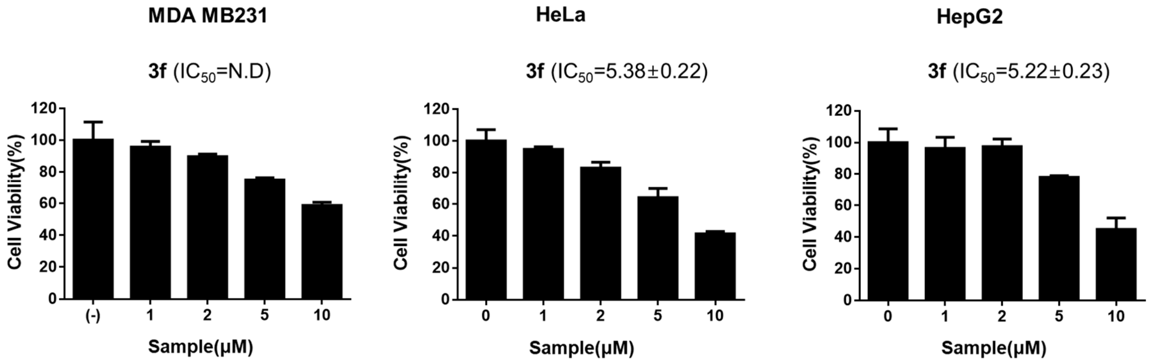

Figure S29. Cell toxicity of 3-[(1S)-1-aminoethyl]-8-(2-methylpyrimidin-5-yl)-2-phenyl-isoquinolin-1-one (**3f**) for MDA-MB 231, HeLa, HepG2.

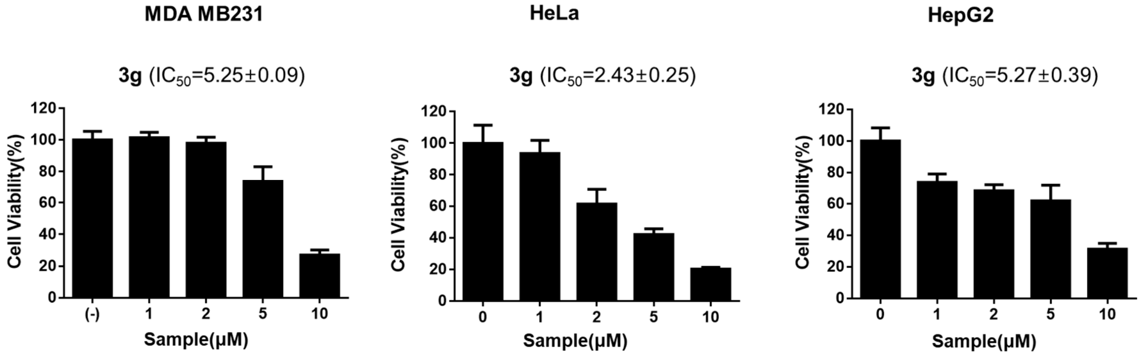

Figure S30. Cell toxicity of 3-[(1*S*)-1-aminoethyl]-8-[2-(1-hydroxy-1-methyl-ethyl)pyrimidin-5-yl]-2-phenyl-isoquinolin-1-one (**3g**) for MDA-MB 231, HeLa, HepG2.

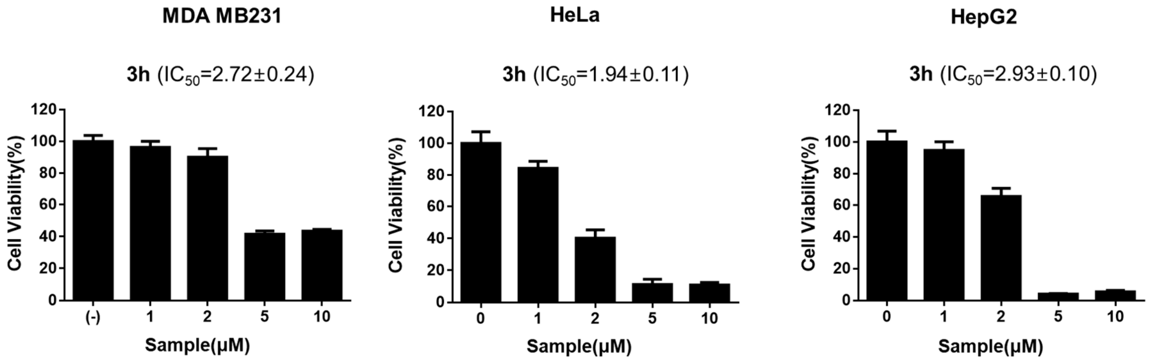

Figure S31. Cell toxicity of 3-[(1*S*)-1-aminoethyl]-8-(2-aminopyrimidin-5-yl)-2-phenyl-isoquinolin-1-one (**3h**) for MDA-MB 231, HeLa, HepG2.

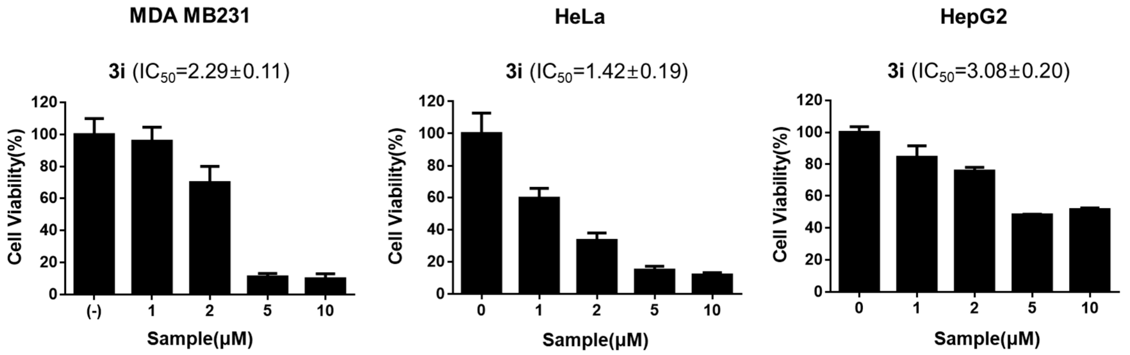

Figure S32. Cell toxicity of 3-[(1*S*)-1-aminoethyl]-8-[2-(dimethylamino)pyrimidin-5-yl]-2-phenyl-isoquinolin-1-one (**3i**) for MDA-MB 231, HeLa, HepG2.

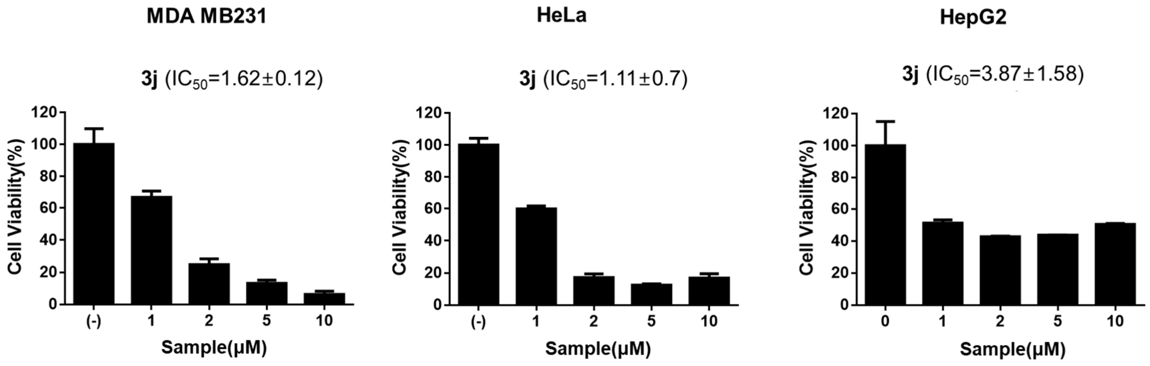

Figure S33. Cell toxicity of 3-[(1*S*)-1-aminoethyl]-2-phenyl-8-[2-(1-piperidyl)pyrimidin-5-yl]isoquinolin-1-one (**3j**) for MDA-MB 231, HeLa, HepG2.

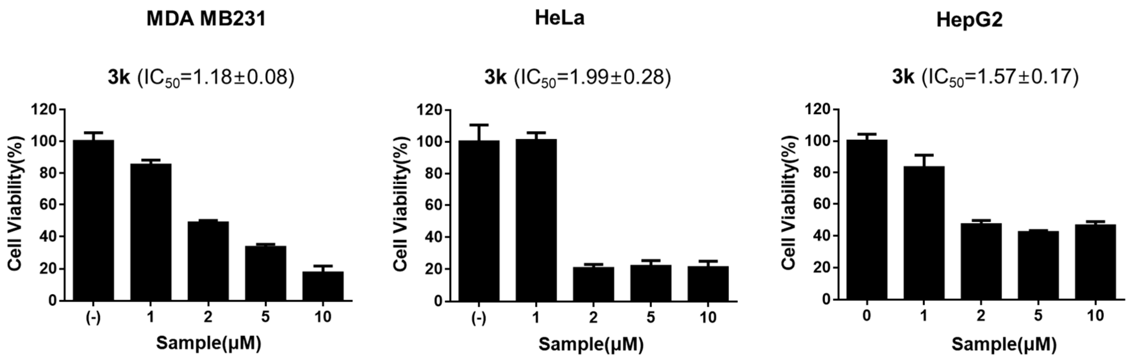

Figure S34. Cell toxicity of 5-[3-[(1*S*)-1-aminoethyl]-1-oxo-2-phenyl-8-isoquinolyl]pyrimidine-2-carbonitrile (**3k**) for MDA-MB 231, HeLa, HepG2.
